# TRIM15 forms a regulatory loop with the AKT/FOXO1 axis and LASP1 to modulate the sensitivity of HCC cells to TKIs

**DOI:** 10.1038/s41419-023-05577-7

**Published:** 2023-01-20

**Authors:** Chong Yang, Xin Jin, Xingchao Liu, Gang Wu, Wenhao Yang, Beichuan Pang, Jipeng Jiang, Dongxu Liao, Yu Zhang

**Affiliations:** 1 Clinical Immunology Translational Medicine Key Laboratory of Sichuan Province & Organ Transplantation Center, Sichuan Provincial People’s Hospital, University of Electronic Science and Technology of China, Chengdu, 611731 Sichuan China; 2 Department of Urology, The Second Xiangya Hospital, Central South University, Changsha, Hunan 410011 China; 3 Uro-Oncology Institute of Central South University, Changsha, Hunan 410011 China; 4 Hunan Key Laboratory of Tumor Models and Individualized Medicine, The Second Xiangya Hospital, Central South University, Changsha, China; 5 Hepatobiliary and Pancreatic Surgery Department, Sichuan Provincial People’s Hospital, University of Electronic Science and Technology of China, Chengdu, 611731 Sichuan China; 6 Chinese Academy of Sciences Sichuan Translational Medicine Research Hospital, Chengdu, 610072 Sichuan China

**Keywords:** Cancer therapy, Oncogenes

## Abstract

For patients with advanced or metastatic Hepatocellular carcinoma (HCC) who are not suitable for surgical resection, systemic therapy has been considered to be the standard treatment. In recent years, a small subset of patients with unresectable HCC have been benefit from tyrosine kinase inhibitors (TKIs), and the overall survival time of these patients is significantly increased. However, all responders ultimately develop resistance to TKI treatment. The tripartite motif (TRIM) family member TRIM15 acts as an E3 ligase to mediate the polyubiquitination of substrates in cells. However, the biological role of TRIM15 in HCC is still an enigma. In our study, our results demonstrated that TRIM15 was abnormally upregulated in liver cancer cells after treated with TKIs and that this upregulation of TRIM15 contributed to TKI resistance in liver cancer cells. Then, we demonstrated that the upregulation of TRIM15 after TKI treatment was mediated by the AKT/FOXO1 axis. Moreover, we demonstrated that TRIM15 induced the nuclear translocation of LASP1 by mediating its K63-linked polyubiquitination, which modulated sensitivity to TKIs by increasing the phosphorylation of AKT and the expression of Snail in liver cancer cells. Collectively, we identified a novel AKT/FOXO1/TRIM15/LASP1 loop in cells, which provided potential candidates for overcoming TKI resistance in HCC.

## Introduction

Hepatocellular carcinoma (HCC) originating from hepatocytes is one of the most common and lethal cancers worldwide [1]. For patients with advanced or metastatic HCC who are not suitable for surgical resection, systemic therapy has been considered to be the standard treatment. In recent years, epidermal growth factor receptor (EGFR) tyrosine kinase inhibitors (TKIs), such as sorafenib and regorafenib, have been approved for the first-line or second-line treatment of advanced HCC [2, 3]. A small subset of patients with unresectable HCC can benefit from these “target” regimens, and the overall survival time of these patients is significantly increased [2, 3]. However, all responders ultimately develop resistance to TKI treatment [4]. Thus, identifying a novel therapeutic strategy to overcome TKI resistance is critical for prolonging the survival of patients with advanced HCC.

The main types of resistance TKI treatment in cancer patients are primary resistance and acquired resistance. The main primary resistance mechanisms include the intrinsic resistance of cancer stem cells to TKIs [5] and resistance caused by EGFR activation. The mechanisms of acquired drug resistance include dysregulation of ATP-binding cassette transporters [6], autophagy [7], epithelial-mesenchymal transition (EMT) [8], tumor microenvironmental factors [9], and epigenetic modifications [9]. Moreover, reactivation of the AKT and ERK signaling pathways is also important for the acquired resistance to TKIs in HCC [10]. Although a number of factors have been reported to participate in the process of TKI resistance, there are still many molecular mechanisms associated with drug resistance that have not yet been identified.

The tripartite motif (TRIM) family member TRIM15 acts as an E3 ligase to mediate the polyubiquitination of substrates in cells [11]. Dysregulation of TRIM15 contributes to tumor progression in pancreatic cancer and non-small cell lung cancer [11, 12]. In this study, we found that TRIM15 was abnormally upregulated in liver cancer cells after treated with TKIs and that this upregulation of TRIM15 contributed to TKI resistance in liver cancer cells. Then, we demonstrated that the upregulation of TRIM15 after TKI treatment was mediated by the AKT/FOXO1 axis. Moreover, we demonstrated that TRIM15 induced the nuclear translocation of LASP1 by mediating its K63-linked polyubiquitination, which modulated sensitivity to TKIs by increasing the phosphorylation of AKT and the expression of Snail in liver cancer cells. Therefore, our results identified an AKT/FOXO1/TRIM15/LASP1 loop and suggested that TRIM15 might be a candidate for improving the sensitivity of HCC to TKIs.

## Methods and materials

### Cell lines, chemicals and antibodies

Liver cancer cell lines Huh7 (#SC0332) and Hep3B (#SC0327) were purchased from the Yuchi Biology (Shanghai, China). All cells were identified by short tandem repeat (STR) profiling. Mycoplasma contamination was regularly detected using the PlasmoTest™ - Mycoplasma Detection Kit (InvivoGen, China). Huh7 and Hep3B cells were cultured in Dulbecco’s modified eagle medium (DMEM) (ThermoFisher Scientific, Shanghai, China) added with 10% fetal bovine serum (FBS) (AC03L055, Shanghai Life-iLab Biotech, China) and incubated at 37 °C in 5% CO_2_. Regorafenib-resistant Huh7 cells and Sorafenib-resistant Huh7 cells were generated as previously reported [13].

Chemicals used as follows: Regorafenib (#S1178, Selleck, China); Sorafenib (#S7397, Selleck, China); AS1842856 (#S8222, Selleck, China); MK2206 (#S1078, Selleck, China); MG132 (#S2619, Selleck, China). The plasmids of TRIM15 used as previously reported [11]. The plasmids of LASP1 were cloning the cDNA of LASP1 into the OmicLinkTM Expression Clone (CMV Promoter) (GeneCopoeia, EX-V0006-M14, USA). The mutant of LASP1 (LASP1 K75A) were also generated by the GeneCopoeia. The siRNA used were obtained from RiboBio (Guangzhou, China). The Annexin V-7AAD Kit (#FXP147-100) was purchased from 4A Biotech, Beijing, China. The shRNAs were purchased from GeneCopoeia (USA). The sequence of siRNA and shRNA was provided in the Table S1.

Antibodies used as follows: Beta Actin (#66009-1-Ig, Proteintech, 1:5000 dilution), TRIM15 (#13623-1-AP, Proteintech, 1:1000 dilution), LASP1 (#68080-1-Ig, Proteintech, 1:1000 dilution), FOXO1 (#2880S, Cell signaling technology,1:500 dilution), PARP1 (#13371-1-AP, Proteintech, 1:1000 dilution), AKT (#bsm-33278M, Bioss antibodies, 1:2000 dilution), S6K1 (#2708, Cell signaling technology,1:1000 dilution), pS6k1-T398 (#9209, Cell signaling technology,1:1000 dilution), cleaved caspase 3 (#9661, Cell signaling technology,1:2000 dilution), Caspase 3 (#19677-1-AP, Proteintech, 1:1000 dilution), pAKT-S473 (#4060S, Cell signaling technology,1:1000 dilution), pAKT-T308 (#13038, Cell signaling technology,1:1000 dilution), cleaved caspase 3 (#9661, Cell signaling technology,1:2000 dilution).

### Co-immunoprecipitation (Co-IP), Western blot analysis and Glutathione S-transferase pull-down assay

The cells were harvested and lyzed. The proteins from the cells were extracted by the RIPA buffer (#P0013, Beyotime, China). The protein amount was quantified by the BCA method. For Co-IP, the proteins were added with protein A + G beads (#P2029, Beyotime, China) and IgG (#A7007, Beyotime, China) or a primary antibody. The mixer was rotated in the cold room overnight. The next day, the beads were washed 6 times with RIPA buffer, and 60 µl of 1× loading buffer was added. Finally, the beads were boiled and subjected to Western blotting.

The procedure of western blot analysis was reported previously [11]. Proteins was boiled and subjected to odium dodecyl sulfatepolyacrylamide gel electrophoresis (SDS-PAGE) gel separation. The proteins were transferred onto 0.45 μm polyvinylidene fluoride membranes (Millipore, USA), and incubated with the corresponding primary antibodies and secondary antibodies. Protein signals were visualized using ECL detection reagent (Thermo Fisher Scientific, USA) and ChemiDoc XRS (Bio-Rad Laboratories, USA).

For Glutathione S-transferase pull-down assay, cells were lysed with 1× RIPA lysis buffer (P0013B, Beyotime, shanghai, China) for 30 min at 4 °C. Glutathione S-transferase (GST) fusion proteins were immobilized on BeyoMag™ Anti-GST Magnetic Beads (P2138, Beyotime, shanghai, China). After washed with 1 × RIPA lysis buffer, the beads were incubated with cell lysates for 4 h. The beads were then washed four times with RIPA lysis buffer and resuspended in loading buffer. The bound proteins were subjected to SDS/PAGE and Western blotting.

### Quantitative real-time PCR (RT-qPCR) and Chromatin immunoprecipitation (ChIP)-qPCR analysis

The details of RT-qPCR were described previously [13]. In brief, TRIzol reagent (Thermo Fisher Scientific, USA) extracted the total RNA from cells. A reverse transcription kit and PCR kit (#RR037A PrimeScript™ RT reagent Kit, #RR430A, TB Green™ Fast qPCR Mix, Takara Bio Inc. Shigo, Japan) was used to performed the RT-qPCR assay. The primer sequences for RT-PCR are provided in Tables S2. Chromatin extraction kit (Abcam, ab117152, USA) and the ChIP Kit Magnetic - One Step (Abcam, ab156907, USA) were applied to performed the ChIP-qPCR. The dilution of antibodies used for ChIP-qPCR was mentioned in the figure legends. The primer sequences for RT-PCR are provided in Tables S3.

### Cell counting Kit-8 (CCK-8) assay

Liver cancer cells were seeded in 96-well plates and approximately 10^4^ cells per well. After culturing for 24 h at 37 °C in 5% CO_2_. 10 μl CCK-8 reagent (#C0037, Beyotime, China) were added into each well and incubated for 1 h under the above conditions, the absorbance at 450 nm were measured by microplate reader.

### In vitro angiogenesis assay

Approximately 1 × 10^5^ HUVEC cells were resuspended with tumor-conditioned supernatant in each well of a 24-well plate containing 250 μl Matrigel (BD Bioscience, USA). Cells were cultured and monitored for tube formation up to 12 h.

### Cell migration and invasion assay

For migration assay, cells were cultured to confluence on six-well plates. The cell layer was scratched and detached cells were removed. For each sample, at least three scratched fields were photographed immediately. Cell migration was evaluated by measuring the cell-covered area. The in vitro cell invasion assay was performed using a BioCoat Matrigel invasion chamber (BD Biosciences) according to the protocol of the manufacturer. Cells were cultured in the insert for 24 h. Cells were fixed in methanol for 15 min and then stained with 1 mg/ml crystal violet for 20 min. At least three fields for each group were photographed after staining, and invaded cells were counted.

### Nude mice xenografts assay

Since the sex of the mice had no effect on the results of the study, we chose half the males and half the females for the experiment. BALB/C-nu/nu mice (Hunan SJA Laboratory Animal Company), 6 weeks old, were individually housed in the animal center of the Sichuan Provincial People’s Hospital. Animals had free access to food and water. The mice were randomly divided into sub-groups. There is no blinding or participants. The liver cancer cells were injected subcutaneously on the left side of the back of mice (5 × 10^6^ cells per mouse). The length and width of the tumor were measured with Vernier caliper every 2 days, and the tumor volume was calculated according to the formula (*L* × *W*^2^)/2. Mice were sacrificed at the appropriate time, and the tumors were collected for further study. Animal experiments were approved by the Ethical Committee on Animal Experiments of the Sichuan Provincial People’s Hospital in Chengdu, China. The animal experiment complied with the National Institutes of Health guide for the care and use of Laboratory animals (NIH Publications No. 8023, revised 1978).

### Statistical analysis

The experimental data were from three independent experiments and presented as the mean ± standard error of the mean (mean ± SEM), GraphPad Prism 5 software was used to calculate the *P* value using unpaired two-sided Student’s t test to compare values between two groups or one-way analysis of variance (ANOVA) followed by Tukey’s multiple comparisons post hoc test to compare values between more than two groups. In all cases, the significance of differences was indicating as follows: *, *P* < 0.05; **, *P* < 0.01; ***, *P* < 0.001; not significant (ns), *P* > 0.05.

## Results

### Regorafenib and sorafenib increased the expression level of TRIM15 in liver cancer cells

**Fig. 1:**
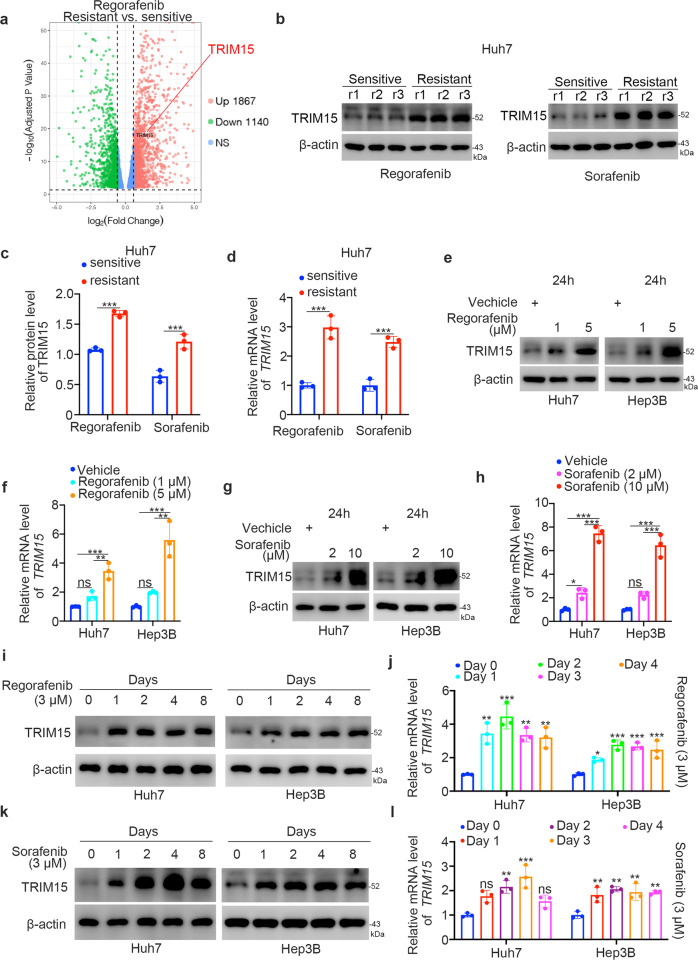
Regorafenib and sorafenib increased the expression level of TRIM15 in liver cancer cells. **a** Analysis of the RNA-seq data of regorafenib-resistant HCC cell lines; **b** Western blot analysis of the whole cell lysate of Huh7 cells. r1, r2, r3 means replication 1, replication 2, replication 3; **c**, **d** RT-qPCR analysis of Huh7 cells. Data presents as mean ± SEM with three replicates. ***, *P* < 0.001. Huh7 and Hep3B cells were treated with indicated dose of regorafenib for 24 h. Cells were harvested for western blot (**e**) and RT-qPCR analysis (**f**). Data presents as mean ± SEM with three replicates. Ns, not significant; **, *P* < 0.01; ***, *P* < 0.001. **g**, **h** Huh7 and Hep3B cells were treated with indicated dose of sorafenib for 24 h. Cells were harvested for western blot (**g**) and RT-qPCR analysis (**h**). Data presents as mean ± SEM with three replicates. Ns not significant; *, *P* < 0.05; ***, *P* < 0.001. **i**, **j** Huh7 and Hep3B cells were treated with 3 μM regorafenib for different time points. Cells were harvested for western blot (**i**) and RT-qPCR analysis (**j**). Data presents as mean ± SEM with three replicates. *, *P* < 0.05; **, *P* < 0.01; ***, *P* < 0.001. **k**, **l** Huh7 and Hep3B cells were treated with 3 μM sorafenib for different time points. Cells were harvested for western blot (**k**) and RT-qPCR analysis (**l**). Data presents as mean ± SEM with three replicates. Ns not significant; *, *P* < 0.05; **, *P* < 0.01.

We previously reported that TRIM15 targets APOA1 for degradation to promote the malignant progression of pancreatic cancer cells [11]. However, the role of TRIM15 in cancer is still unknown. Tyrosine kinase inhibitors are considered to be the first-line therapy for patients with advanced or metastatic HCC. Of note, transcriptome analysis [14] demonstrated that TRIM15 was abnormally upregulated in regorafenib-resistant HCC cell lines (Fig. 1a). Then, we established the regorafenib-resistant and sorafenib-resistant Huh7 cells and the characteristics of these cells were shown in Supplementary Fig. 1a, b. We found that the protein and mRNA levels of TRIM15 were elevated in regorafenib- and sorafenib-resistant Huh7 cells compared to cells sensitive to these inhibitors (Fig. 1b–d). Then, we showed that regorafenib and sorafenib increased the protein and mRNA levels of TRIM15 in a dose-dependent manner in both Huh7 and Hep3B cells (Fig. 1e–h). Moreover, the regulation of TRIM15 by treatment with a constant concentration of regorafenib or sorafenib was enhanced with increasing drug exposure time in both Huh7 and Hep3B cells. Thus, these data indicated that TKI treatment increases the expression of TRIM15 in HCC cells.

### The AKT/FOXO1 axis is the key mediator of TKI-induced TRIM15 overexpression in liver cancer cells

**Fig. 2:**
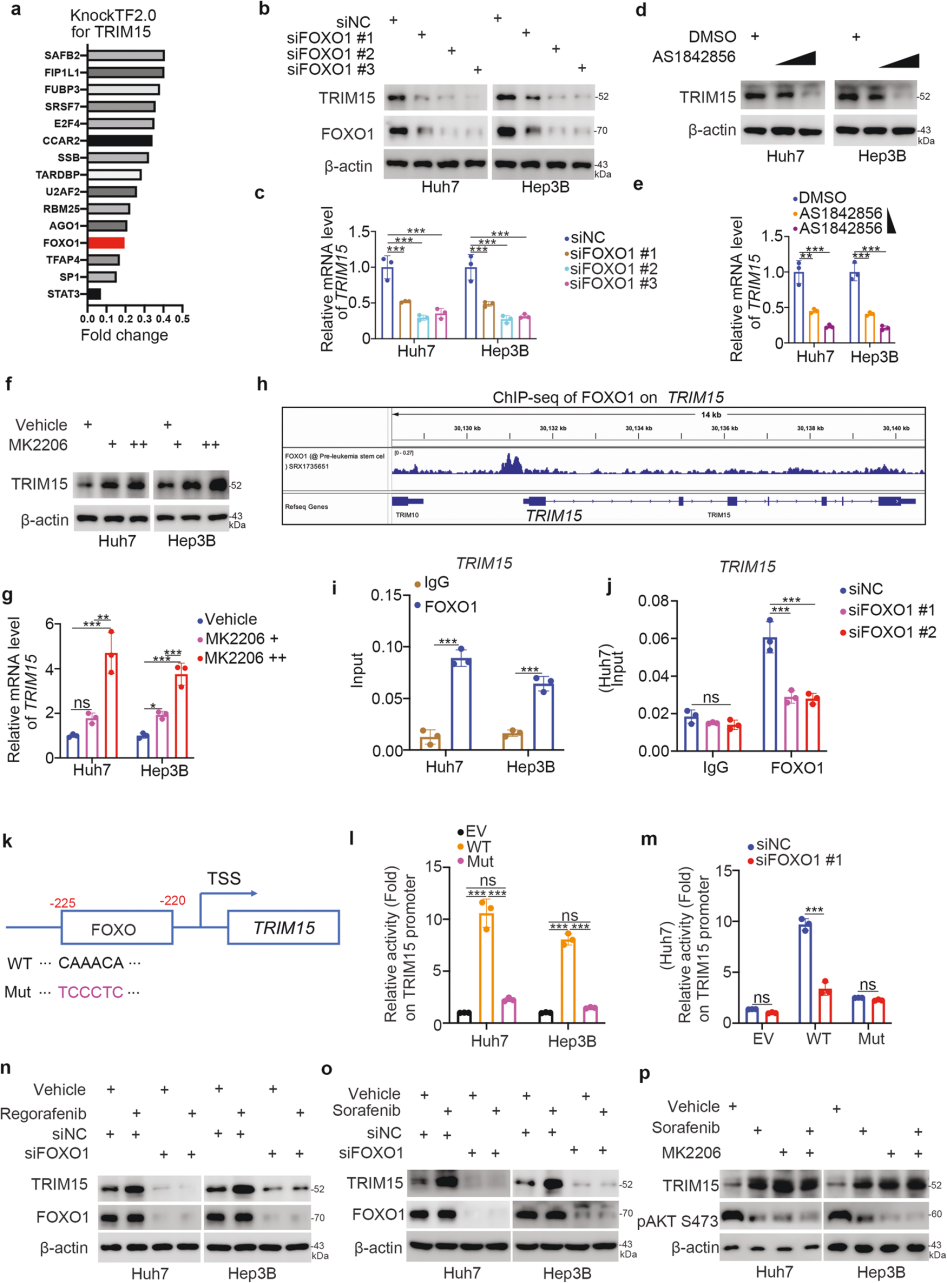
The AKT/FOXO1 axis is the key mediator of TKI-induced TRIM15 overexpression in liver cancer cells. **a** Analysis of the KnockTF2.0 database web sites to find the potential transcriptional factors of TRIM15; **b**, **c** Huh7 and Hep3B cells were infected with indicated siRNAs for 48 h. Cells were harvested for western blot (**b**) and RT-qPCR analysis (**c**). Data presents as mean ± SEM with three replicates. ***, *P* < 0.001. **d**, **e** Huh7 and Hep3B cells were treated with DMSO, 200 nM AS1842856, 1 μM AS1842856 for 24 h. Cells were harvested for western blot (**d**) and RT-qPCR analysis (**e**). Data presents as mean ± SEM with three replicates. **, *P* < 0.01; ***, *P* < 0.001. **f**, **g** Huh7 and Hep3B cells were treated with DMSO, 1 μM MK2206, 5 μM MK2206 for 24 h. Cells were harvested for western blot (**f**) and RT-qPCR analysis (**g**). Data presents as mean ± SEM with three replicates. **, *P* < 0.01; ***, *P* < 0.001. **h** the ChIP-seq of FOXO1 on the promoter region of *TRIM15*. IGV v2.9.0 was used for analysis and visualization of ChIP-seq data. **i** the ChIP-qPCR analysis by using the IgG (Dilution 1:100) or FOXO1 (Dilution 1:50) antibodies in Huh7 and Hep3B cell. Data presents as mean ± SEM with three replicates. ***, *P* < 0.001. **j** Huh7 cells were transfected with indicated siRNAs for 48 h. Cells were harvested for ChIP-qPCR analysis by using the IgG or FOXO1 antibodies. Data presents as mean ± SEM with three replicates. Ns not significant; ***, *P* < 0.001. **k** diagram showed the sequence and position of FOXO element on the promoter region of *TRIM15*. TSS, transcriptional start site; WT, wild type; Mut, mutant type. **l** Huh7 and Hep3B cells were transfected with empty vector (EV), GV592-TRIM15 plasmids WT or Mut for 24 h. Cells were harvested and activity of TRIM15 promoter were measured. Data presents as mean ± SEM with three replicates. Ns not significant; ***, *P* < 0.001. **m** Huh7 cells were transfected with indicated siRNAs for 48 h. Then, cells were were transfected with empty vector (EV), GV592-TRIM15 plasmids WT or Mut for 24 h. Cells were harvested and activity of TRIM15 promoter were measured. Data presents as mean ± SEM with three replicates. Ns not significant; ***, *P* < 0.001. **n** Huh7 and Hep3B cells were transfected with indicated siRNAs for 48 h. Cells were treated with or without 5 μM regorafenib for 24 h. Cells were harvested for western blot analysis. **o** Huh7 and Hep3B cells were transfected with indicated siRNAs for 48 h. Cells were treated with or without 5 μM sorafenib for 24 h. Cells were harvested for western blot analysis. **p** Huh7 and Hep3B cells were treated with vehicle, 5 μM sorafenib, 5 μM MK2206, or 5 μM sorafenib plus 5 μM MK2206 for 24 h. Cells were harvested for western blot analysis.

To understand why TRIM15 is upregulated after TKI treatment in HCC cells, we used the online KnockTF2.0 database (http://www.licpathway.net/KnockTF/search.php) to predict the potential transcription factors for TRIM15 (Fig. 2a) and use ChIP-Atlas database (http://chip-atlas.org/) to investigate whether these transcription factors bound to the promoter region of *TRIM15*. The KnockTF2.0 database showed a variety of transcriptional factors, such as SAFB2, FIP1L1, FUBP2, or FOXO1, could be the transcriptional factors for TRIM15. Meanwhile, ChIP-Atlas database demonstrated that FOXO1 bound to the promoter region of TRIM15. In addition, FOXO1 silencing by siRNA transfection was found to decrease the protein and mRNA levels of TRIM15 in both Huh7 and Hep3B cells (Fig. 2b, c). Then, we also demonstrated that treatment with FOXO1 inhibitors downregulated TRIM15 expression in liver cancer cells (Fig. 2d, e). We showed that the PI3K/AKT inhibitors, including MK2206 or LY294002, treatment increased the expression of TRIM15 in both Huh7 and Hep3B cells (Fig. 2f, g and Supplementary Fig. 2a). We also showed that MK2206 or LY294002 treatment, which eliminated the phosphorylation of AKT, led to the cell apoptosis and reducing the cell proliferation in HCC cells (Supplementary Fig. 2b–d). It has been reported that AKT phosphorylates FOXO1 to prevent its nuclear translocation [15]. In addition, sorafenib treatment decreased the phosphorylation level of AKT in cancer cells. ChIP-seq data of FOXO1 from the ChIP-Atlas database showed that FOXO1 could bind to the promoter region of *TRIM15* (Fig. 2h). The subsequent ChIP‒qPCR assay with IgG and an anti-FOXO1 antibody confirmed that FOXO1 can bind to the promoter region of *TRIM15* in Huh7 and Hep3B cells (Fig. 2i). Inhibition of FOXO1 decreased the binding of FOXO1 to the *TRIM15* promoter (Fig. 2j). In addition, we analyzed the promoter region of TRIM15 and found that it contained a FOXO1 consensus binding motif (Fig. 2k). We cloned the DNA sequences for the FOXO1 binding region and a FOXO1 binding region mutant to generate two GV592-TRIM15 promoter plasmids. The empty vector (EV), GV592-TRIM15 WT or GV592-TRIM15 Mut was transfected into Huh7 and Hep3B cells (Fig. 2l). We found that the luciferase activity of the WT TRIM15 promoter was greater than that of the Mut promoter or the EV in both Huh7 and Hep3B cells (Fig. 2l). We also showed that knockdown of FOXO1 decreased the luciferase activity of the WT TRIM15 promoter in Huh7 cells but had no effect on the luciferase activity of the Mut TRIM15 promoter (Fig. 2m). Moreover, we demonstrated that FOXO1 silencing attenuated the increase in TRIM15 expression induced by regorafenib or sorafenib treatment in liver cancer cells (Fig. 2n, o). Furthermore, AKT inhibitor treatment diminished the change in TRIM15 expression mediated by sorafenib (Fig. 2p). Taken together, our data suggested that TKI-induced upregulation of TRIM15 is mediated by the AKT/FOXO1 axis in liver cancer cells.

### Abnormally upregulated TRIM15 leads to TKI resistance in HCC

**Fig. 3:**
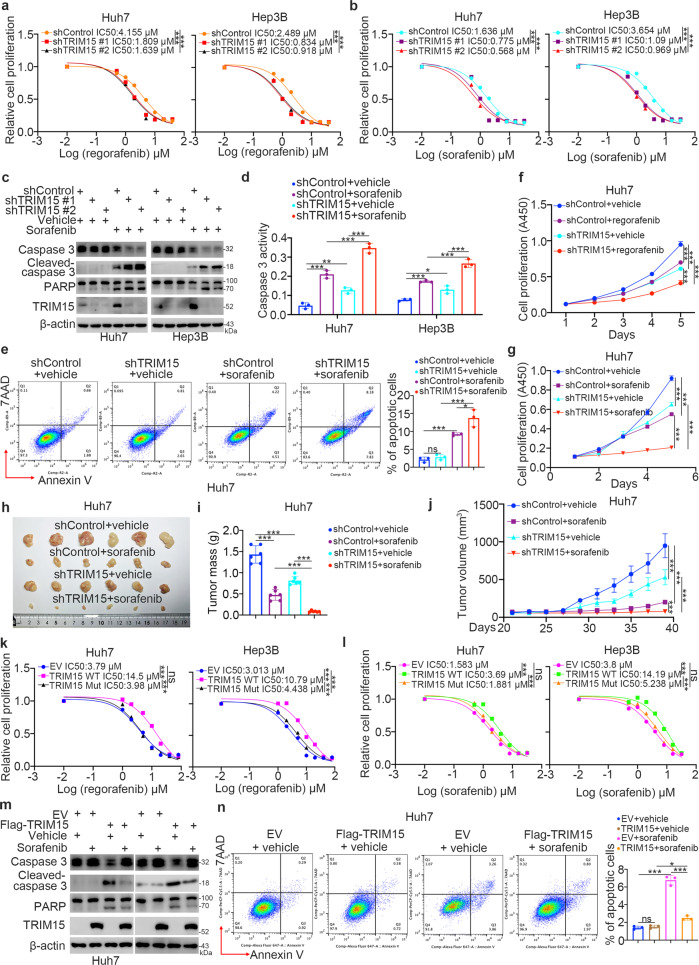
Abnormally upregulated TRIM15 leads to TKI resistance in HCC. **a** Huh7 and Hep3B cells were infected with indicated shRNAs for 48 h. Cells were treated with a serial dose of regorafenib for 24 h. Then, cells were collected for CCK-8 assay. *N* = 3, ***, *P* < 0.001. **b** Huh7 and Hep3B cells were infected with indicated shRNAs for 48 h. Cells were treated with a serial dose of sorafenib for 24 h. Then, cells were collected for CCK-8 assay. *N* = 3, ***, *P* < 0.001. **c**–**e** Huh7 and Hep3B cells were infected with indicated shRNAs for 48 h. Cells were treated with 3 μM sorafenib for 24 h. Cells were harvested for western blot (**c**), caspase 3 activity (**d**), and Annexin V-7AAD assay (**e**). Data presents as mean ± SEM with three replicates. Ns not significant; *, *P* < 0.05; **, *P* < 0.01; ***, *P* < 0.001. **f**, **g** Huh7 cells were infected with indicated shRNAs for 48 h. Cells were treated with vehicle or 3 μM regorafenib or sorafenib, and these cells were subjected to CCK-8 assay. Data presents as mean ± SEM with three replicates. ***, *P* < 0.001. **h**–**j** Huh7 cell were infected with indicated shRNAs for 72 h. After puromycin selection, cells were subcutaneously injected into the nude mice. The tumor image was shown in panel (**h**), the tumor mass was shown in panel (**i**), and tumor growth curve was shown in panel (**j**). Data presents as mean ± SEM with six replicates. ***, *P* < 0.001. **k** Huh7 and Hep3B cells were transfected with EV, Flag-TRIM15 WT, or Flag-TRIM15 Mut (delta Ring domain) for 48 h. Cells were treated with a serial dose of regorafenib for 24 h. Then, cells were collected for CCK-8 assay. *N* = 3, ***, *P* < 0.001. **l** Huh7 and Hep3B cells were transfected with EV, Flag-TRIM15 WT, or Flag-TRIM15 Mut (delta Ring domain) for 48 h. Cells were treated with a serial dose of sorafenib for 24 h. Then, cells were collected for CCK-8 assay. *N* = 3, ***, *P* < 0.001. **m**, **n** Huh7 and Hep3B cells were transfected with EV or Flag-TRIM15 WT for 48 h. Cells were treated with 3 μM sorafenib for 24 h. Cells were harvested for western blot (**m**) and Annexin V-7AAD assay (**n**). Data presents as mean ± SEM with three replicates. Ns not significant; *, *P* < 0.05; ***, *P* < 0.001.

Since TRIM15 was upregulated in TKI-resistant liver cancer cells, we sought to determine whether TRIM15 participates in modulating the sensitivity of HCC cells to TKIs. To this end, Huh7 and Hep3B liver cancer cells were treated with serial concentrations of regorafenib or sorafenib after TRIM15 knockdown (Fig. 3a, b). TRIM15 silencing decreased the IC50 values of regorafenib and sorafenib in these liver cancer cells (Fig. 3a, b). Moreover, we also demonstrated that knockdown of TRIM15 significantly decreased the IC50 values of TKI in TKI-resistant cells (Supplementary Fig. 3a–c). Then, we demonstrated that TRIM15 knockdown enhanced apoptosis induced by sorafenib treatment in Huh7 and Hep3B cells (Fig. 3c–e). Then, the results of in vitro and in vivo cell proliferation assays indicated that inhibition of TRIM15 expression enhanced the antitumor effect of TKIs in liver cancer cells (Fig. 3f–j). In contrast, overexpression of wild-type (WT) TRIM15 but not the catalytically dead TRIM15 mutant (Mut) [11] increased the IC50 values of TKIs in Huh7 and Hep3B cells (Fig. 3k, l). In addition, we found that TRIM15 overexpression prevented sorafenib-induced apoptosis in liver cancer cells (Fig. 3m, n). Together, these data indicated that TRIM15 is responsible for modulating the sensitivity of HCC cells to sorafenib.

### TRIM15 activates the AKT-mTOR signaling pathway and promotes proliferation and EMT in HCC

**Fig. 4:**
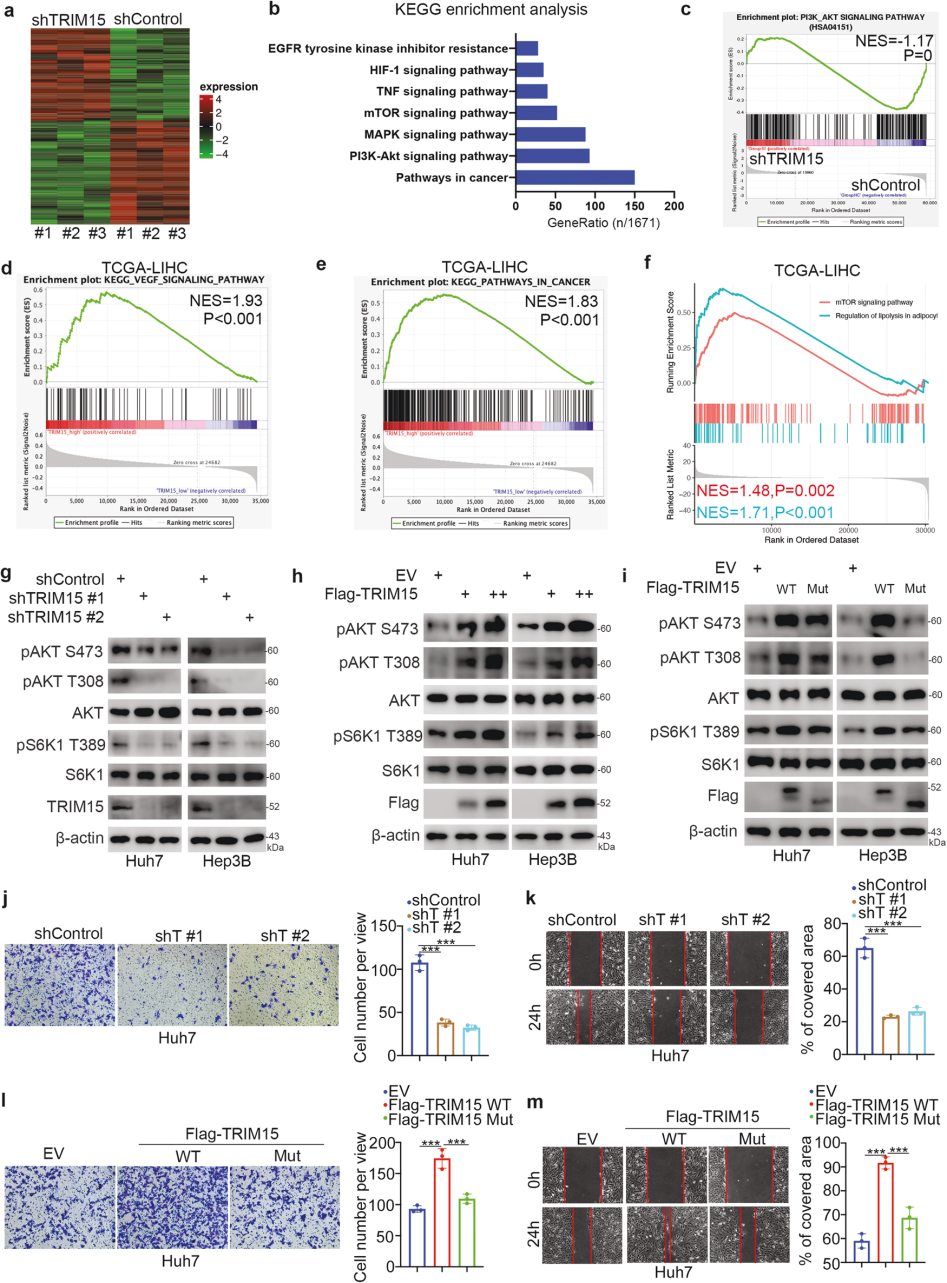
TRIM15 activates the AKT-mTOR signaling pathway and promotes EMT in HCC. **a**–**c** Huh7 cell were infected with indicated shRNAs for 72 h. After puromycin selection, cells were harvested for RNA-seq analysis. The heatmap of RNA-seq was shown in panel (**a**), KEGG enrichment analysis was shown in panel (**b**), and GSEA analysis was shown in panel (**c**). **d**–**f** GSEA analysis was performed by the using the TCGA-KIRC dataset. **g** Huh7 and Hep3B cells were infected with indicated shRNAs for 72 h. Cells were harvested for western blot analysis. **h** Huh7 and Hep3B cells were transfected with EV, 2 ng Flag-TRIM15 or 5 ng Flag-TRIM15 for 24 h. Cells were harvested for western blot analysis. **i** Huh7 and Hep3B cells were transfected with indicated plasmids for 24 h. Cells were harvested for western blot analysis. **j**, **k** Huh7 cells were infected with indicated shRNAs for 72 h. Cells were harvested for transwell assay and migration assay. Data presents as mean ± SEM with three replicates. ***, *P* < 0.001. **l**, **m** Huh7 cells were transfected with indicated plasmids for 24 h. Cells were harvested for transwell assay and migration assay. Data presents as mean ± SEM with three replicates. ***, *P* < 0.001.

To explore the mechanism by which TRIM15 regulates sensitivity to TKIs in HCC, transcriptome analysis was performed after TRIM15 knockdown in Huh7 cells (Fig. 4a). Kyoto Encyclopedia of Genes and Genomes (KEGG) enrichment analysis and gene set enrichment analysis (GSEA) of the RNA-seq data indicated that TRIM15 was associated with multiple cancer-related pathways, including pathways in cancer, the PI3K-AKT signaling pathway, the MAPK signaling pathway, the mTOR signaling pathway, the TNF signaling pathway, the HIF-1 signaling pathway, and the EGFR kinase inhibitor resistance pathway (Fig. 4b, c). In addition, KEGG enrichment and GSEA analyses of the TCGA-KIRC dataset suggested that TRIM15 was closely associated with the VEGF signaling pathway, cancer-related pathways, and the mTOR signaling pathway (Fig. 4d–f). In addition, we found that TRIM15 might be involved in the regulation of lipolysis in adipocytes, which was consistent with our previous finding that TRIM15 targets APOA1 for degradation in pancreatic cancer [11] (Fig. 4f).

Then, we showed that TRIM15 knockdown decreased the phosphorylation levels of AKT and S6K1 in both Huh7 and Hep3B cells (Fig. 4g). In contrast, overexpression of wild-type TRIM15 but not the catalytically dead mutant activated AKT-mTOR signaling in liver cancer cells (Fig. 4h, i). Moreover, the xenografts in Fig. 3h were subjected to detect TRIM15 or pAKT-S473 via western blot analysis (Supplementary Fig. 3d). We also noticed that knockdown of TRIM15 or treatment of sorafenib decreased the phosphorylation of AKT on Ser-473 in the tumor xenograft. And treatment of sorafenib on TRIM15 depletion cells decreased more pAKT-S473 in Supplementary Fig. 3d. In addition, we analyzed the RNA-seq data comparing the difference between regorafenib-resistant and -sensitive HCC cells reported by Sofer S et al. [14]. (Supplementary Fig. 4a). We found that the activation of PI3K/AKT pathway was involved in the TKI resistance in HCC (Supplementary Fig. 4a), which is consistent with the literature reported [16]. We simultaneously analyzed the RNA-seq results of TRIM15 and the sequencing results of TKI resistant cells and found that there were three genes, *PGF, GNG4, THBS4*, associated with the PI3K-AKT signaling pathway were changed after TRIM15 depletion or in regorafenib resistant HCC cells (Supplementary Fig. 4b). Then, we showed that knockdown of TRIM15 decreased the mRNA level of PGF in Huh7 cells (Supplementary Fig. 4c), and overexpression of TRIM15 increased the expression of PGF in Huh7 cells (Supplementary Fig. 4d). In addition, we found that PGF was upregulated in the regorafenib-resistant cells compared to regorafenib-sensitive cells (Supplementary Fig. 4e).

Since the AKT-mTOR axis plays an important role in modulating epithelial-mesenchymal- transition, which is the key player in the modulation of TKI resistance in tumor cells [17–20], and we previously reported that TRIM15 contributes to EMT and metastasis in pancreatic cancer [11], we investigated whether TRIM15 regulates the process of EMT in liver cancer cells. Similar to previous observations, we found that TRIM15 knockdown inhibited the invasion and migration of Huh7 cells (Fig. 4j, k). However, overexpression of TRIM15 WT but not the catalytically dead mutant promoted the invasion and migration of Huh7 cells (Fig. 4l, m). Furthermore, we knocked down TRIM15 in Huh7 cells, and these cells were pre-treated with MK2206 or LY294002 to block the PI3K/AKT signaling pathway. We demonstrated that inhibition of the AKT pathway diminished the change of cell proliferation and cell migration abilities induced by depletion of TRIM15 in Huh 7 cells (Supplementary Fig. 4f–i). Then, we also showed that overexpressed the TRIM15 WT or dead mut plasmids induced the change of Huh7 cell proliferation ability could be attenuated by the pre-treatment with MK2206 or LY294002 (Supplementary Fig. 4J, k). Moreover, MK2206 treatment could diminished the effect of overexpression of TRIM15 WT on the cell migration (Supplementary Fig. 4l). Thus, these data suggested that TRIM15 contributes to the activation of the AKT-mTOR pathway and the promotion of proliferation and EMT in liver cancer cells.

### TRIM15 interacts with LASP1 to regulate sensitivity to TKIs in HCC

**Fig. 5:**
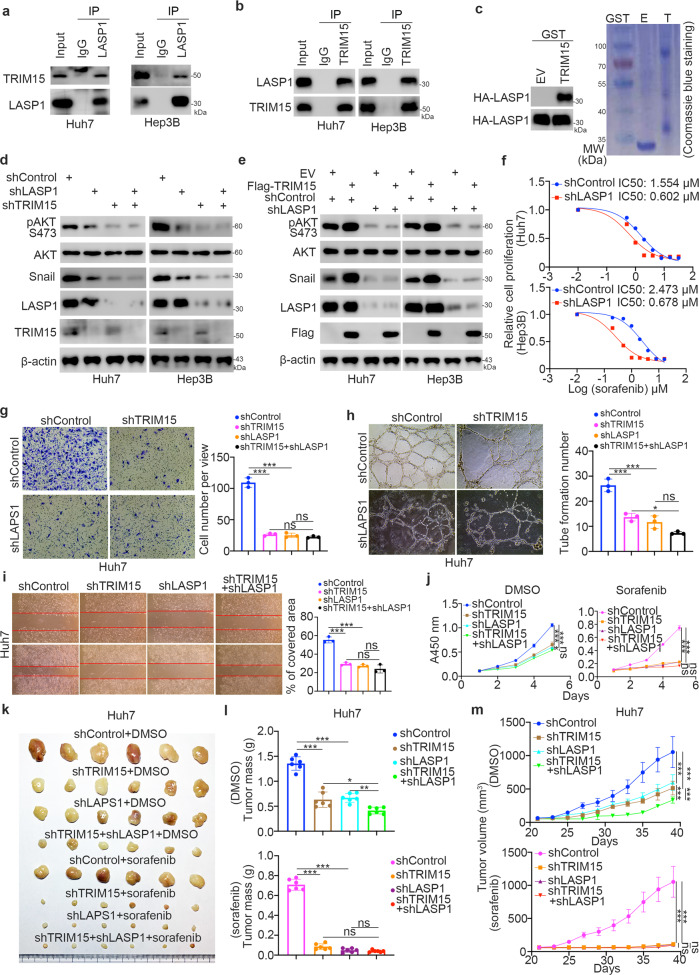
TRIM15 interacts with LASP1 to regulate sensitivity to TKIs in HCC. **a**, **b** The whole cell lysate of Huh7 and Hep3B were collected. The Co-IP was performed by using the IgG, TRIM15 or LASP1 antibodies. **c** The HA-LASP1 plasmids were transfected into the Huh7 cells. After 48 h, cells were harvested and subjected to GST-pull down assay. **d** Huh7 and Hep3B cells were infected with indicated shRNAs for 72 h. Cells were harvested for western blot analysis. **e** Huh7 and Hep3B cells were infected with indicated shRNAs. After 48 h, cells were transfected with plasmids for 24 h. Cells were harvested for western blot analysis. **f** Huh7 and Hep3B cells were infected with indicated shRNAs for 72 h. Cells were harvested and treated with a serial dose of sorafenib for 24 h. Then, these cells were collected for CCK-8 assay. *N* = 3, ***, *P* < 0.001. **g**–**i** Huh7 and Hep3B cells were infected with indicated shRNAs. After 72 h, cells were harvested for transwell assay (**g**), angiogenesis assay (**h**), and migration assay (**i**). Data presents as mean ± SEM with three replicates. Ns not significant; *, *P* < 0.05; ***, *P* < 0.001. **j**–**m** Huh7 cells were infected with indicated shRNAs for 72 h. After puromycin selection, cells were collected and treated with or without sorafenib (3 μM sorafenib for cells, 50 mg/kg oral administration for nude mice) for CCK-8 assay or nude mice xenograft assay. The tumor image was shown in panel (**k**), the tumor mass in panel (**l**), and the growth curve in panel (**m**). For CCK-8 assay, data presents as mean ± SEM with three replicates. Ns not significant; ***, *P* < 0.001. For xenograft assay, data presents as mean ± SEM with three replicates. Ns not significant; *, *P* < 0.05; **, *P* < 0.01; ***, *P* < 0.001.

In this part, we sought to determine how TRIM15 activates AKT signaling and promotes EMT in liver cancer cells. After rechecking the previously reported mass spectrometry data for TRIM15, we found that LASP1 might be the binding partner of TRIM15. It has been reported that LASP1 induces the phosphorylation of FAK-AKT pathway components and negatively regulates PTEN expression in tumor cells [21, 22], which contributes to hyperactivation of the AKT pathway. Also, a study reported that LASP1 increases the expression of snail to induce EMT in lung cancer [23]. Thus, we examined whether LASP1 is the key mediator of TRIM15-induced TKI resistance in liver cancer cells. First, endogenous coimmunoprecipitation using anti-TRIM15 or anti-LASP1 antibodies was performed in Huh7 and Hep3B cells. We found that TRIM15 interacted with LASP1 in liver cancer cells (Fig. 5a, b). Moreover, the interaction between TRIM15 and LASP1 was confirmed by confocal microscopy in Huh7 cells (Supplementary Fig. 5a). In addition, the GST pulldown assay demonstrated that TRIM15 bound to LASP1 in vitro (Fig. 5c). Mechanically, we demonstrated that LASP1 interacted with the C-terminal recombinant of TRIM15 via the GST-pull down assay (Supplementary Fig. 5b). Then, we showed that knockdown of LASP1 attenuated the changes in the phosphorylation of AKT and the expression level of snail induced by TRIM15 inhibition or overexpression (Fig. 5d, e). In addition, we showed that knockdown of LASP1 decreased the IC50 values of sorafenib in Huh7 and Hep3B cells (Fig. 5f). Moreover, we demonstrated that LASP1 silencing diminished the effects of TRIM15 knockdown on EMT and angiogenesis in Huh7 cells (Fig. 5g–i). Furthermore, the results of in vitro and in vivo cell proliferation assays showed that combined knockdown of TRIM15 and LASP1 did not further sensitize Huh7 cells to sorafenib (Fig. 5j–m). Moreover, we revealed that overexpression of TRIM15 did not further promote the cell invasion, migration, angiogenesis, or cell proliferation capability in combination with knockdown of LASP1 in Huh7 cells (Supplementary Fig. 5c–f). And overexpressed TRIM15 also did not affect the sensitivity of Huh7 cells to sorafenib (Supplementary Fig. 5f). Together, our data suggested that LASP1 is the key mediator through which TRIM15 modulates sorafenib sensitivity in liver cancer cells.

### TRIM15 promotes K63-linked ubiquitination of LASP1 in liver cancer cells

**Fig. 6:**
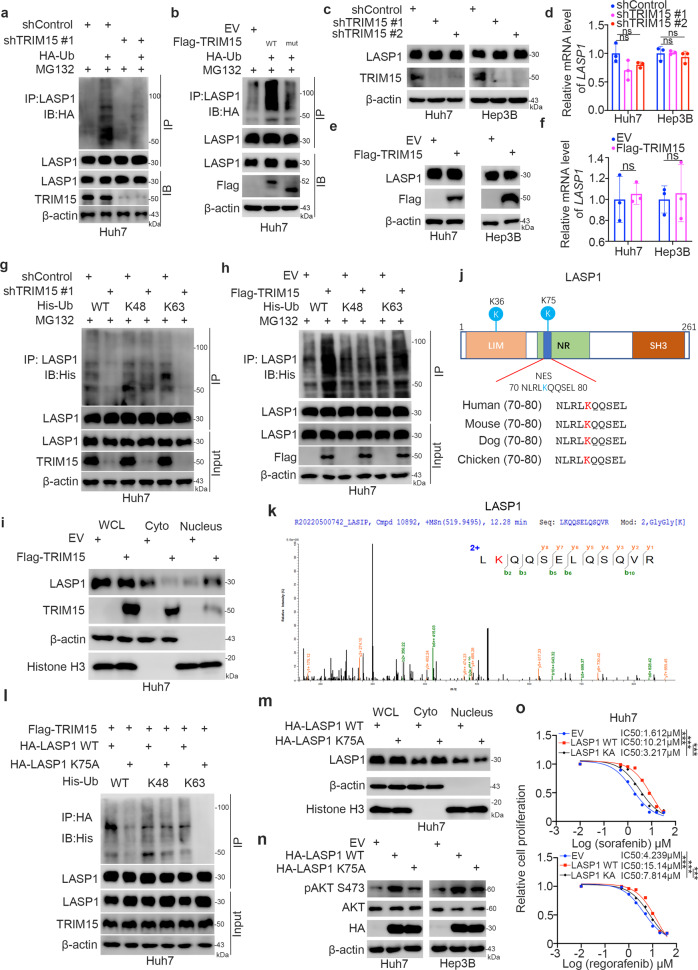
TRIM15 promotes K63-linked ubiquitination of LASP1 in liver cancer cells. **a** Huh7 cells were infected with indicated shRNAs for 48 h. Cells were transfected with or without HA-Ub for 24 h. Then, these cells were harvested for co-IP and western blot analysis. **b** Huh7 cells were transfected with indicated plasmids for 48 h. Then, these cells were harvested for co-IP and western blot analysis. **c**, **d** Huh7 and Hep3B cells were infected with indicated shRNAs for 48 h. Then, these cells were harvested for western blot (**c**) and RT-qPCR assay (**d**). Data presents as mean ± SEM with three replicates. Ns, not significant. **e**, **f** Huh7 and Hep3B cells were infected with indicated plasmids for 48 h. Then, these cells were harvested for western blot (**e**) and RT-qPCR assay (**f**). Data presents as mean ± SEM with three replicates. Ns, not significant. **g**, Huh7 cells were infected with indicated shRNAs for 48 h. Cells were transfected with indicated plasmids for 24 h. Then, these cells were harvested for co-IP and western blot analysis. **h** Huh7 cells were transfected with indicated plasmids for 48 h. Then, these cells were harvested for co-IP and western blot analysis. **i** Huh7 cells were transfected with indicated plasmids for 48 h. Then, these cells were harvested for western blot analysis. **j** A schematic model depicting that the nuclear exporting sequence (NES) and the ubiquitination site of LASP1. **k** The K75 site of ubiquitination site of LASP1. **l** Huh7 cells were transfected with indicated plasmids for 48 h. Then, these cells were harvested for co-IP and western blot analysis. **m** Huh7 cells were transfected with indicated plasmids for 48 h. Then, these cells were harvested for western blot analysis. **n** Huh7 cells were transfected with indicated plasmids for 48 h. Then, these cells were harvested for western blot analysis. **o** Huh7 cells were transfected with indicated plasmids for 48 h. Then, these cells were treated with a serial dose of sorafenib or regorafenib for 24. Cells were harvested for CCK-8 assay. *N* = 3, ***, *P* < 0.001.

Since TRIM15 is known to be an E3 ligase, we investigated whether TRIM15 regulates the ubiquitination of LASP1 in cells. First, we showed that TRIM15 knockdown decreased the ubiquitination of LASP1 in Huh7 cells (Fig. 6a). In contrast, we demonstrated that overexpression of TRIM15 WT but not the catalytically dead mutant promoted the ubiquitination of LASP1 in liver cancer cells (Fig. 6b). In addition, genuine endogenous ubiquitination assay showed that inhibition of TRIM15 decreased the ubiquitination of LASP1 in Huh7 cells (Supplementary Fig. 6a). Interestingly, neither knockdown nor overexpression of TRIM15 affected the protein or mRNA level of LASP1 in Huh7 and Hep3B cells (Fig. 6c–f). We have previously reported that TRIM15 functioned as an E3 ligase to promote the ubiquitination and degradation of APOA1 [11]. In addition, Zhu et al. has been mentioned that TRIM15 mediates the K63-linked ubiquitination of ERK1/2 that increases the phosphorylation of ERK1/2 [24]. Meanwhile, Zhu et al. also showed that inhibition of TRIM15 did not change the total protein level of ERK1/2 [24]. Similarly, we found that knockdown of TRIM15 decreased the total ubiquitination and K63-linked ubiquitination but not K48-linked ubiquitination of LASP1 in liver cancer cells (Fig. 6g). In contrast, TRIM15 overexpression enhanced K63-linked ubiquitination of LASP1 in Huh7 cells (Fig. 6h). Literatures have documented that K63 linked ubiquitination is also generally known to accumulate by treating lysosome inhibitors [25]. Although MG132 is known as a proteasomal inhibitor, it also inhibits cathepsin K, which make MG132 inhibit both the proteasome and lysosome pathway [25, 26]. We have tested the effect of lysosome inhibitors on the K-63 ubiquitination of LASP1 (Supplementary Fig. 6b). We showed that the lysosome inhibitor (chloroquine) increased the K63 linked ubiquitination of LASP1 mediated by TRIM15 (Supplementary Fig. 6b). Given that TKI treatment increased the TRIM15 expression in HCC cells, we would like test whether TKI made effect on the changes of ubiquitination of LASP1 in HCC cells. It is not surprising that sorafenib treatment increased the total ubiquitination or K63-linked ubiquitination of LASP1 in Huh7 cells (Supplementary Fig. 6c). It has been reported that K63-linked ubiquitination participates in regulating the subcellular localization of target proteins [27]. Similarly, we found that overexpression of TRIM15 induced the nuclear translocation of LASP1 in Huh7 cells (Fig. 6i and Supplementary Fig. 6d). Then, we analyzed ubiquitinated LASP1 by mass spectrometry to identify the potential ubiquitination sites in LASP1. Two ubiquitination sites (lysines 36 and 75, K36 and K75) in LASP1 were identified (Fig. 6j). Notably, K75 is located in the previously reported nuclear export signal (amino acids 71-77) [28] (Fig. 6j, k). We constructed a K75A mutant of LASP1 to prevent its ubiquitination. We showed that TRIM15 overexpression did not further increase K63-linked ubiquitination of the LASP1 K75A mutant in Huh7 cells (Fig. 6l). Then, we also demonstrated that compared to wild-type LASP1, the LASP1 K75A mutant was preferentially retained in the cytosol (Fig. 6m). Thus, our results indicated that TRIM15 regulates the nuclear translocation of LASP1 in liver cancer cells. It has been mentioned that the nuclear localization of LASP1 activates AKT signaling and upregulates snail expression [23, 29]. We showed that overexpression of LASP1 ΔNES mutant made little effect on increasing the phosphorylation of AKT compared to LASP1 WT in both Huh7 and Hep3B cells (Supplementary Fig. 6e). Then, we demonstrated that the LASP1 K75A mutant had a weaker effect on increasing the phosphorylation of AKT than WT LASP1 in Huh7 and Hep3B cells (Fig. 6n). Furthermore, we showed that the LASP1 K75A mutation had little effect on the IC50 values of sorafenib in liver cancer cells (Fig. 6o). Meanwhile, we have compared the FOXO1/TRIM15/LASP1 axis between TKI-sensitive and -resistant cells. We demonstrated that the phosphorylation level of pAKT S473 and pFOXO1 S319 and the protein level of TRIM15 were upregulated in TKI-resistant cells compared with TKI-sensitive cells (Supplementary Fig. 6f, i). Then, we also showed that the nucleus portion of LASP1 in TKI-resistant cells was more than that in TKI-sensitive cells (Supplementary Fig. 6g, j). Moreover, we revealed that the sorafenib inhibition effect on inactivating the AKT/FOXO1 pathway was diminished in TKI-resistant cells compared to TKI-sensitive cells (Supplementary Fig. 6h). In addition, we have detected the FOXO1/TRIM15/LASP1/AKT loop in tumor samples from HCC patients with TKI sensitive or resistance (Supplementary Fig. 6k, l). We demonstrated that the expression level of pAKT-S473, pFOXO1-S319, and TRIM15 were up-regulated in sorafenib resistant tumor samples from HCC patients compared to those in sorafenib sensitive group (Supplementary Fig. 6k). Meanwhile, we also showed that the nucleus portion of LASP1 in sorafenib resistant group were more than those in sorafenib sensitive group (Supplementary Fig. 6l). Moreover, we demonstrated that there were positive correlations between pAKT S473 and pFOXO1 S319, pAKT S473 and TRIM15, pFOXO1 S319 and TRIM15, TRIM15 and nucleus portion of LASP1, nucleus portion of LASP1 and pAKT S473 (Supplementary Fig. 6m). We also showed that there was a negative correlation between TRIM15 and cytoplasmic portion of LASP1 (Supplementary Fig. 6m). Although these data reveal the correlation among the FOXO1/TRIM15/LASP1/AKT loop, many correlations are not statistically significant. This may be due to insufficient sample size (*N* = 8). Taken together, these data suggested that the ubiquitination of LASP1 mediated by TRIM15 promoted LASP1 nuclear translocation to activate the AKT signaling pathway, which was responsible for inducing sorafenib resistance in liver cancer cells.

## Discussion

**Fig. 7:**
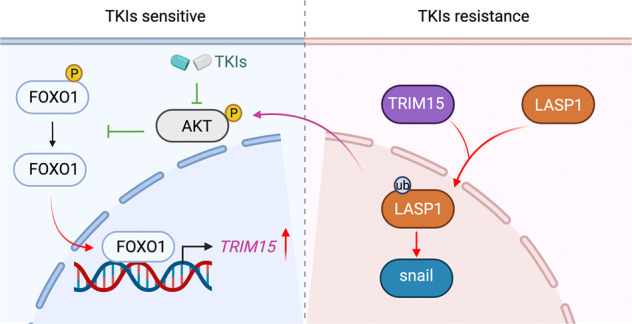
A hypothesis model depicting that TKI treatment increased the expression of TRIM15 by inhibiting the AKT/FOXO1 axis in HCC. The upregulated TRIM15 contributed to TKI resistance by promoting the ubiquitination and nuclear localization of LASP1, which activated the AKT signaling pathway and increased the snail expression in HCC.

It has been reported that TKIs such as sorafenib inhibit the MAPK pathway in HCC cells [30]. Inhibition of the MAPK pathway leads to compensatory activation of the AKT signaling pathway, which is commonly seen in patients with sorafenib-resistant HCC [16]. The AKT signaling pathway is essential for apoptosis and drug resistance in tumor cells, and inhibiting the AKT axis could enhance sensitivity to sorafenib in HCC [31]. Moreover, epithelial-mesenchymal transition (EMT) induced by long-term exposure to sorafenib treatment is the major cause of sorafenib resistance in HCC [32]. Transcription factors, including snail, act as molecular switches for EMT in cells [33]. The literature indicates that snail maintains sorafenib resistance in HCC [34]. Additionally, snail is a downstream target of the AKT/GSK3β signaling pathway in cancer cells [35, 36]. In this study, we showed that TRIM15 interacted with LASP1 and promoted its nuclear translocation. Nuclear LASP1 is known to activate the AKT signaling pathway and increase the expression of snail [23, 29]. In addition, we showed that FOXO1 regulated the expression of TRIM15 after TKI treatment. In addition, we previously reported that FOXO1 was the key mediator linking the MAPK and AKT signaling pathways in tumor cells [37]. Therefore, we identified an AKT/FOXO1/TRIM15/LASP1 loop in HCC (Fig. 7), which might contribute to TKI resistance in HCC. Of note, we noticed that TKI treatment almost eliminates p-AKT and increased the TRIM15 expression in HCC cell without TKI resistance (Fig. 2). But this is the result with short-term TKIs treatment. It has been documented that re-activation of PI3K/AKT pathway is a common feature for acquired TKI, such as sorafenib, resistant cancer cells [16, 38, 39]. As we known, acquired resistance to TKI treatment is long term process. Here, the mechanism study was divided into two parts. The first part elucidated that TRIM15 decreased the sensitivity of sorafenib via activating the LASP1/AKT or LASP1/snail axis in HCC cells. The second part showed that the AKT/FOXO1 axis regulated the expression of TRIM15 in HCC cells. There is a negative feedback loop between AKT and TRIM15, and TKIs treatment could break down this feedback loop. We thought the effect of TKI treatment on decreasing the p-AKT was very strong at first. Despite secondary elevation of TRIM15, it is not sufficient to counteract the inhibitory effect of TKIs on AKT in a short time, which is the reason why TKI has a good antitumor effect in the early stage of drug administration. We believe that the regulatory function of TRIM15 on AKT has been gradually shown over time which is one of the causes of secondary resistance to TKIs in HCC cells.

TRIM15 is dysregulated in tumor cells and associated with tumorigenesis. TRIM15 is a TNF-α-induced E3 ligase [40]. Here, we showed that TKI treatment inhibited AKT signaling, which enhanced the binding of FOXO1 to the promoter region of *TRIM15*. However, the cancer-related role of TRIM15 in HCC is controversial. TRIM15 was found to be overexpressed in non-small cell lung cancer and to correlate with unfavorable patient prognoses [41]. Additionally, inhibiting TRIM15 suppressed the proliferation, migration and invasion of esophageal squamous cell carcinoma cells [42]. In contrast, TRIM15 was found to be downregulated in colon and gastric cancers and to exert an antitumor effect by inhibiting cell migration and invasion [11]. Tissue specificity may account for these different biological functions of TRIM15. As an E3 ligase, TRIM15 promotes the ubiquitination of substrates to regulate cellular processes. We previously reported that TRIM15 promotes APOA1 ubiquitination and degradation in pancreatic cancer [11]. Liang et al. reported that TRIM15 directly targets Keap1 for degradation [12]. In addition to enhancing the degradation of its substrates, TRIM15 has also been reported to mediate K63-linked ubiquitination of its substrates, such as TAK1 [40] and ERK1/2 [24], and modulate the function of these proteins. Similarly, we demonstrated that TRIM15 promoted K63-linked ubiquitination of LASP1 to regulate the subcellular localization of LASP1 in HCC cells. The mass spectrometry and RNA-seq data for TRIM15 indicated that TRIM1 might target other substrates and be involved in other pathways. Thus, the role of TRIM15 needs to be further studied.

Our results demonstrated that TKI treatment increased the expression of TRIM15 by inhibiting the AKT/FOXO1 axis in HCC cells. Then, we showed that TRIM15 upregulation contributed to TKI resistance in HCC. The underlying mechanism might be that TRIM15 interacted with LASP1 to promote its nuclear localization, which activated the AKT signaling pathway and EMT. Thus, we identified an AKT/FOXO1/TRIM15/LASP1 loop in cells, which provided potential candidates for overcoming TKI resistance in HCC.

## Supplementary information

Supplementary information

Original Data File

aj-checklist

## Data Availability

All data generated or analyzed during this study are included in this published article.
