# Supplementary material for: TRIM15 forms a regulatory loop with the AKT/FOXO1 axis and LASP1 to modulate the sensitivity of HCC cells to TKIs
Source: Cell Death Dis. 2023 Jan 20;14(1):47. doi: 10.1038/s41419-023-05577-7 (PMC9859813; doi:10.1038/s41419-023-05577-7)

**TRIM15 forms a regulatory loop with the AKT/FOXO1 axis and LASP1 to modulate the sensitivity of HCC cells to TKIs**

Chong Yang, Xin Jin, Xingchao Liu, Gang Wu, Wenhao Yang, Beichuan Pang, Jipeng Jiang, Dongxu Liao, Yu Zhang

**Supplementary methods and materials**

**RNA sequencing and analysis**

A total of 1 µg of RNA per sample was used as the starting material for RNA sequencing (RNA-seq). RNA integrity was assessed using the RNA Nano 6000 Assay Kit of the Bioanalyzer 2100 system (Agilent Technologies, CA, USA). Clean data (clean reads) were obtained by removing reads containing adapter, reads containing ploy-N and low quality reads from raw data. At the same time, Q20, Q30 and GC content the clean data were calculated. All the downstream analyses were based on the clean data with high quality. Sequencing libraries were generated using the NEBNext Ultra RNA Library Prep Kit for Illumina (NEB, USA) following the manufacturer’s instructions, and index codes were added to attribute sequences to each sample. Clustering of the samples was performed on the cBot Cluster Generation System using the TruSeq PE Cluster Kit v3-cBot-HS (Illumina) according to the manufacturer’s instructions. After cluster generation, libraries were sequenced on an Illumina Novaseq platform, and 150-bp paired-end reads were generated. FeatureCounts v1.5.0-p3 was used to count the read numbers mapped to each gene. Differential expression analysis was performed using the DESeq2 R package (1.16.1), and the cluster Profiler R package was used to test the statistical enrichment of differentially expressed genes (DEGs) in KEGG (Kyoto Encyclopedia of Genes and Genomes) pathways. Three replicates were performed in each group. The RNA-seq data has deposited in the GEO dataset (GSE216001).

**Collection of clinical specimens**

Clinical specimens of HCC were collected from t Hepatobiliary and Pancreatic Surgery Department, Sichuan Provincial People’s Hospital, University of Electronic Science and Technology of China. Ethical approval for the use of human tissues (HCC cancer patients with or without sorafenib resistance) was obtained by the local ethics committee (Sichuan Provincial People’s Hospital, China). Written informed consent was acquired from all patients before surgery. We collected the specimens from patients with HCC and underwent a resection of the tumor following sorafenib therapy. Postoperative imaging examination, such as computed tomography, was used to evaluate the therapeutic effect of sunitinib therapy. According to the response evaluation criteria in solid tumors (RECIST) version 1.1 (24,25), we defined those patients achieved complete remission or partial remission as sorafenib sensitive, and patients with progressive disease (PD) as sorafenib resistance.

**Immunofluorescence assay**

Cells were ﬁxed in 4% paraformaldehyde for 15 min. After washed in PBS three times, ﬁxed cells were permeabilized with 0.2% Triton X-100 for 20 min, washed in PBS and then blocked in PBS supplemented with 10% goat serum. Cells were incubated with indicated primary antibody at 4℃ overnight. After washed three times with PBS, cells were incubated with secondary antibody that was conjugated with Alexa Fluor 488 dye or Alexa Fluor 594 dye (Thermo Fisher Scientiﬁc) for 1 h at room temperature. After washed three times with PBS, cells were counterstained with DAPI (4’, 6-diamidino-2-phenylindole).


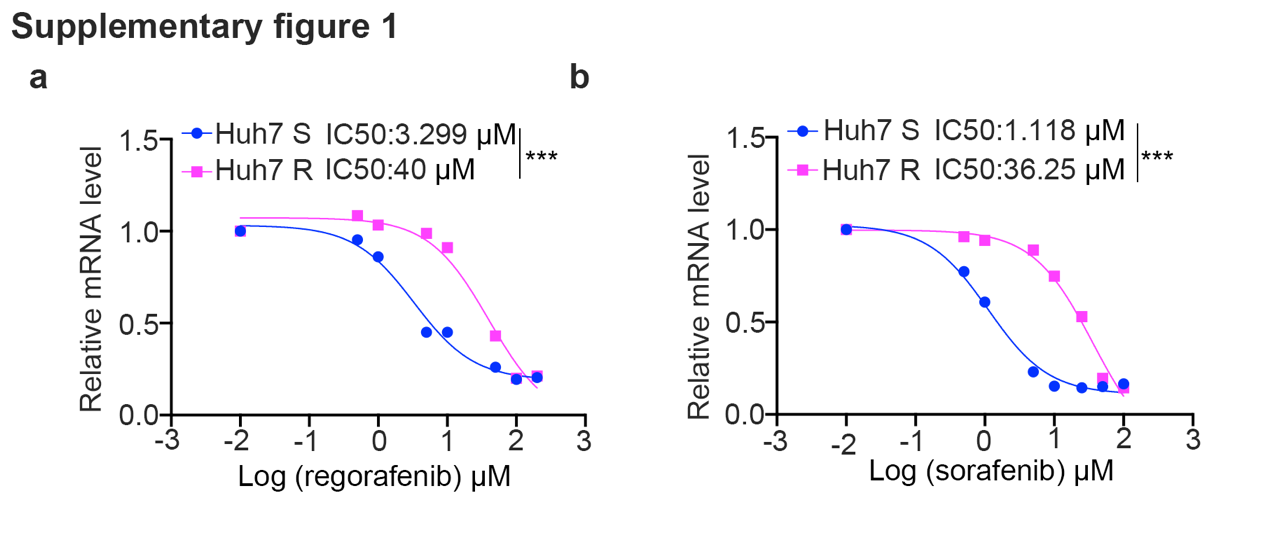


**Supplementary figure 1. a,** The Huh7 cells with regorafenib sensitive (Huh7 S) or resistant (Huh7 R) were treated with a serial dose of regorafenib for 24 h. Then, cells were collected for CCK-8 assay. The IC50 values were indicated in the panel. N = 3, ***, P < 0.001. **b,** The Huh7 cells with sorafenib sensitive (Huh7 S) or resistant (Huh7 R) were treated with a serial dose of sorafenib for 24 h. Then, cells were collected for CCK-8 assay. The IC50 values were indicated in the panel. N = 3, ***, P < 0.001.

**
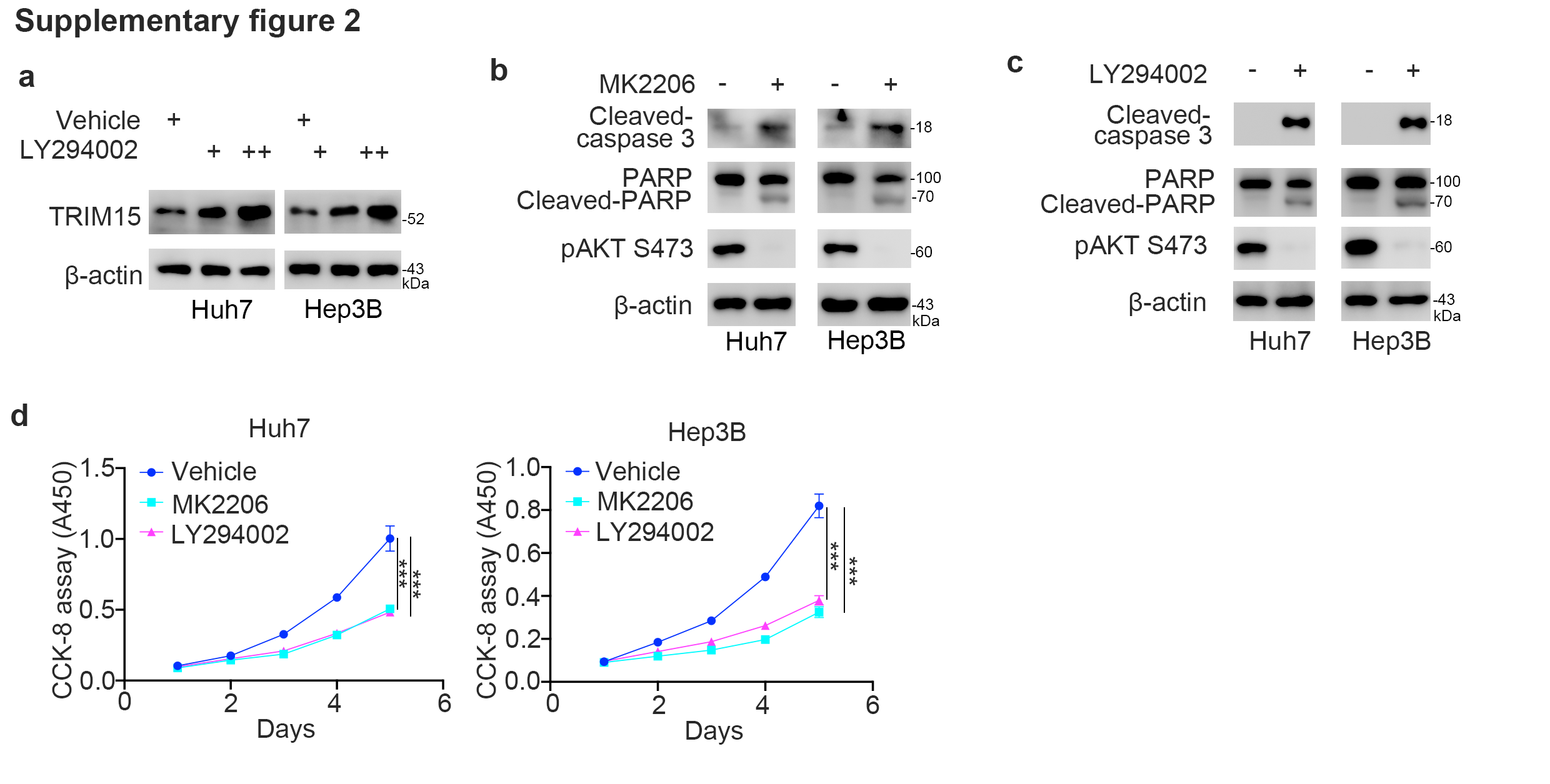
**

**Supplementary figure 2. a,** Huh7 and Hep3B cells were treated with Vehicle, 1 μM (+) LY294002 or 10 μM (++) LY294002 for 24 h. Cells were harvested for western blot analysis. **b**, Huh7 and Hep3B cells were treated with Vehicle, or 10 μM MK2206 for 24 h. Cells were harvested for western blot analysis. **c**, Huh7 and Hep3B cells were treated with Vehicle, or 10 μM LY294002 for 24 h. Cells were harvested for western blot analysis. **d**, Huh7 and Hep3B cells were treated with Vehicle, 10 μM MK2206, or 10 μM LY294002. Cells were harvested for CCK-8 assay. Data presents as mean ± SEM with three replicates. P < 0.001.

**
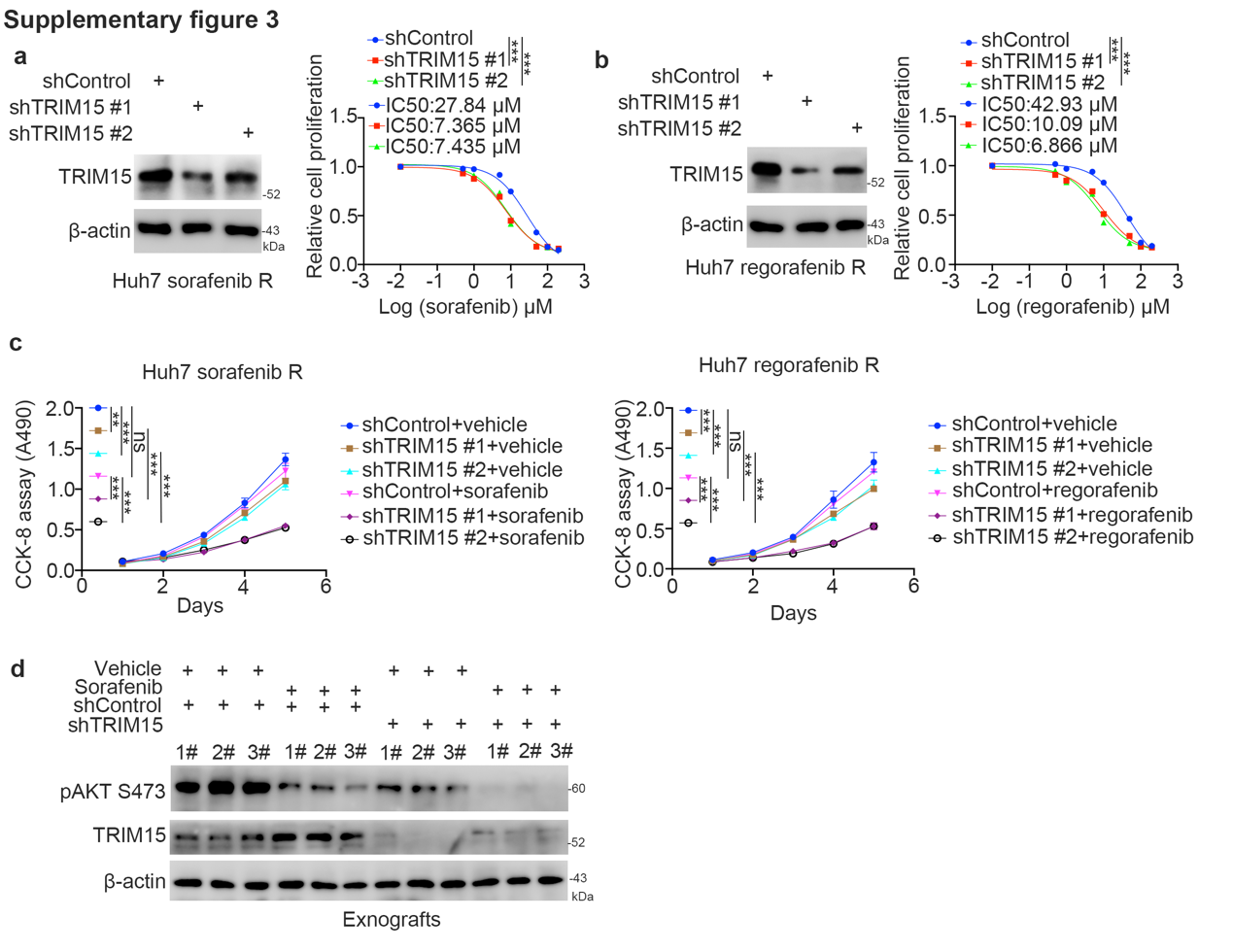
**

**Supplementary figure 3.** **a**, Huh7 sorafenib resistant cell lines were infected with indicated shRNAs for 72h. Cells were harvested for western blot analysis or CCK-8 assay after treated with as serial dose of sorafenib. N = 3, ***, P < 0.001. **b**, Huh7 regorafenib resistant cell lines were infected with indicated shRNAs for 72h. Cells were harvested for western blot analysis or CCK-8 assay after treated with as serial dose of regorafenib. N = 3, ***, P < 0.001. **c**, Huh7 sorafenib resistant cell lines were infected with indicated shRNAs for 72h. Cells were treated with vehicle or sorafenib (20 μM) and subjected to CCK-8 assay. Huh7 regorafenib resistant cell lines were infected with indicated shRNAs for 72h. Cells were treated with vehicle or regorafenib (20 μM) and subjected to CCK-8 assay. Data presents as mean ± SEM with three replicates. Ns, not significant; **, P < 0.01; ***, P < 0.001. **d**, the xenograft from the Fig. 3h were subjected to western blot analysis. N = 3.


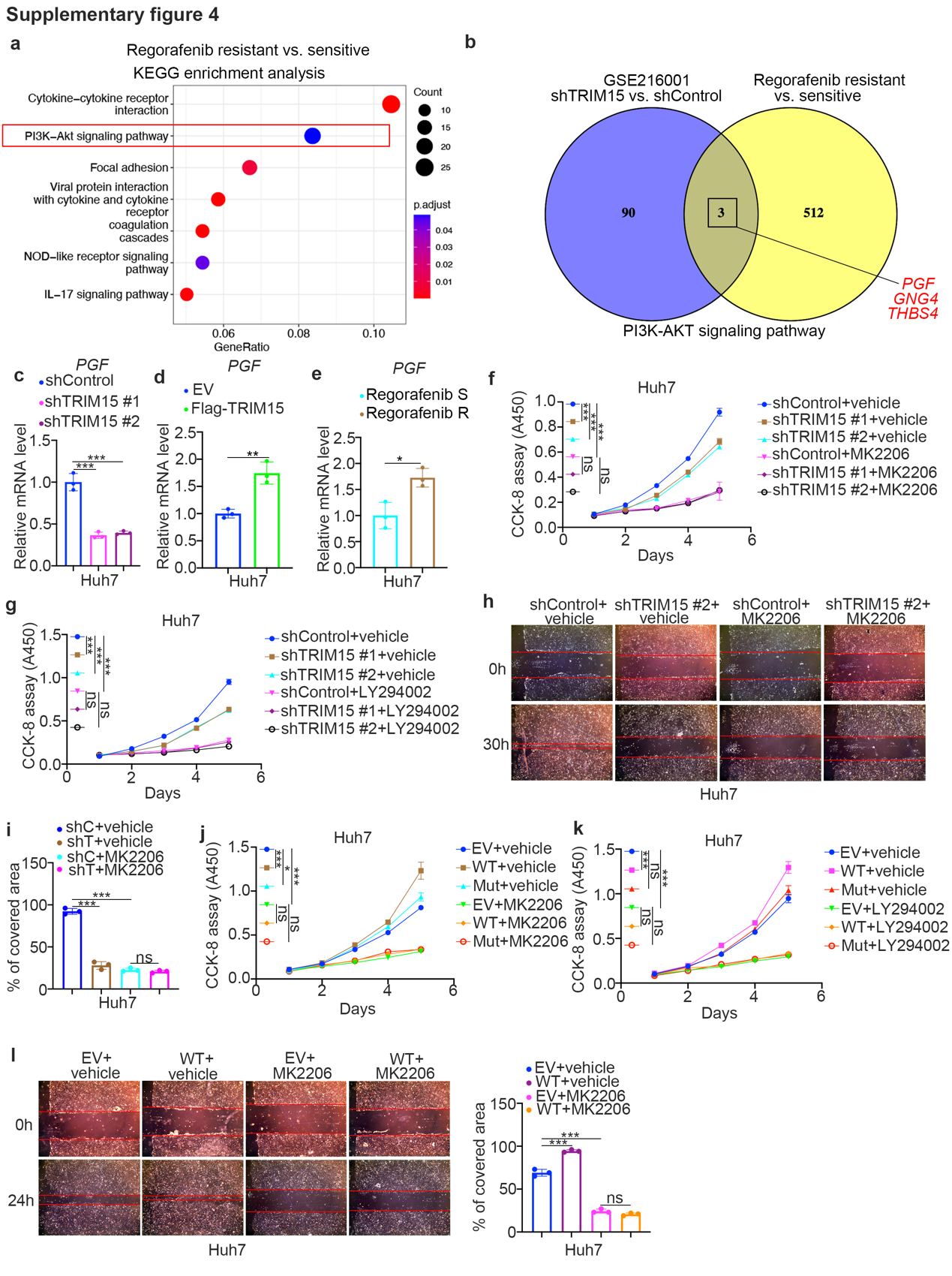


**Supplementary figure 4. a,** KEGG analysis of RNA-seq reported by Sofer S et al. **b**, the Venn map showed the co-regulated genes of GES216001 datasets or datasets reported by Sofer S et al. **c**, Huh7 cells were infected with indicated shRNAs for 72 h. Cells were harvested for RT-qPCR analysis. Data presents as mean ± SEM with three replicates. ***, P < 0.001. **d**, Huh7 cells were transfected with indicated plasmids for 48 h. Cells were harvested for RT-qPCR analysis. Data presents as mean ± SEM with three replicates. **, P < 0.01. **e**, The Huh7 cells with regorafenib sensitive (Huh7 S) or resistant (Huh7 R) were harvested for RT-qPCR analysis. Data presents as mean ± SEM with three replicates. *, P < 0.05. **f**, Huh7 cells were infected with indicated shRNAs for 72 h. Then, cells were treated with or without MK2206 (10 μM) and subjected to CCK-8 assay. Data presents as mean ± SEM with three replicates. Ns, not significant; ***, P < 0.001. **g**, Huh7 cells were infected with indicated shRNAs for 72 h. Then, cells were treated with or without LY294002 (10 μM) and subjected to CCK-8 assay. Data presents as mean ± SEM with three replicates. Ns, not significant; ***, P < 0.001. **h and i**, Huh7 cells were infected with indicated shRNAs for 72 h. Then, cells were treated with or without MK2206 (10 μM) and subjected to wound healing assay. Data presents as mean ± SEM with three replicates. Ns, not significant; ***, P < 0.001. **j**, Huh7 cells were transfected with indicated plasmids for 48 h. Then, cells were treated with or without MK2206 (10 μM) and subjected to CCK-8 assay. Data presents as mean ± SEM with three replicates. Ns, not significant; *, P < 0.05; ***, P < 0.001. **k**, Huh7 cells were transfected with indicated plasmids for 48 h. Then, cells were treated with or without LY294002 (10 μM) and subjected to CCK-8 assay. Data presents as mean ± SEM with three replicates. Ns, not significant; ***, P < 0.001. **i**, Huh7 cells were transfected with indicated plasmids for 48 h. Then, cells were treated with or without MK2206 (10 μM) and subjected to wound healing assay. Data presents as mean ± SEM with three replicates. Ns, not significant; *, P < 0.05; ***, P < 0.001.

**
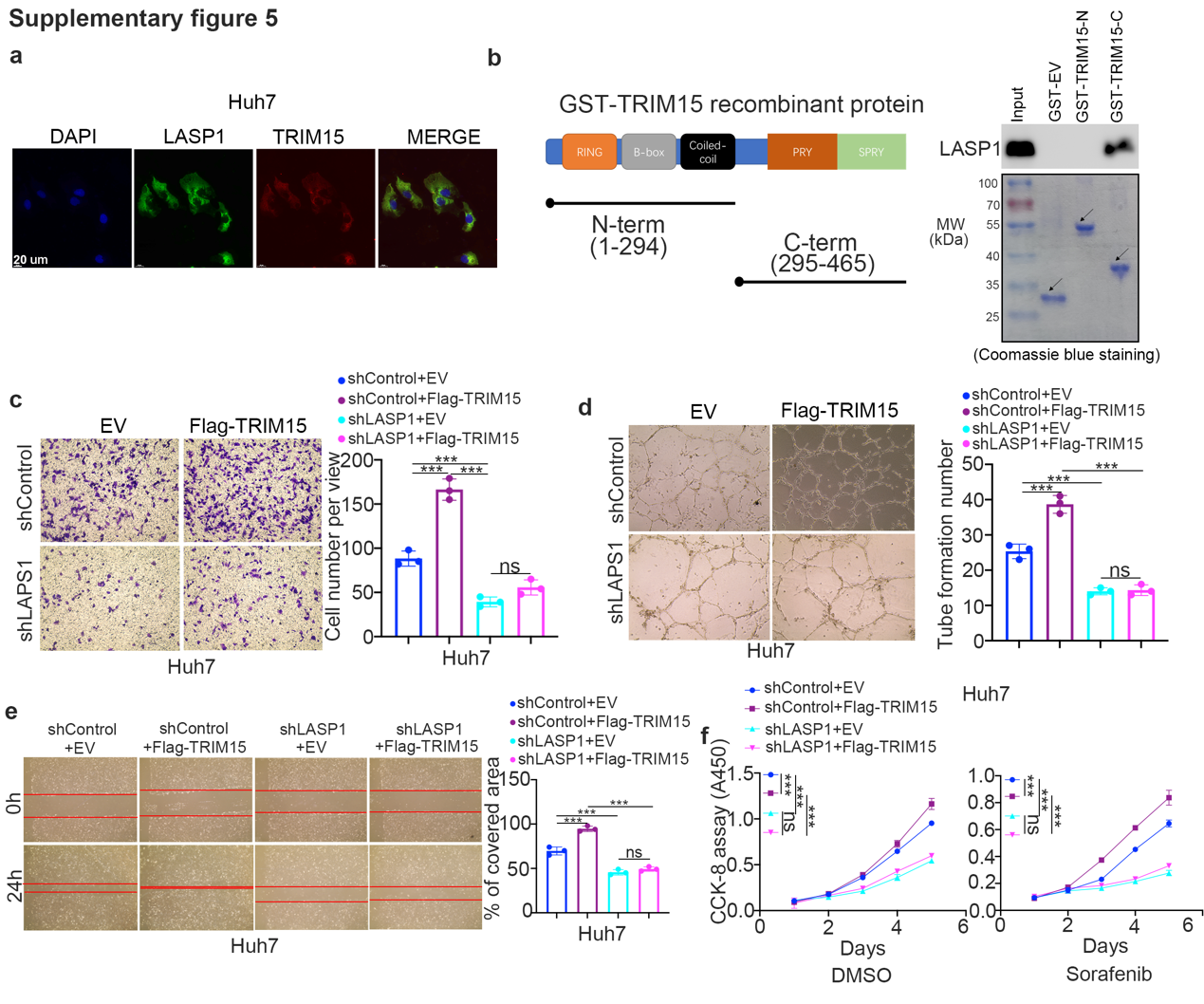
**

**Supplementary figure 5. a,** Huh7 cells were stained with DAPI, LASP1 antibody, TRIM15 antibody and subjected to immunofluorescence assay. **b**, the schematic diagram depicted two GST-TRIM15 recombinant constructs according to the sequence of TRIM15. The LASP1 protein in Huh7 whole cell lysate was pulled down by GST-TRIM15 recombinant protein. **c-f**, Huh7 cell were infected with indicated shRNAs for 48h. Then, cells were transfected with indicated plasmids for another 24h. These cells were harvested for transwell assay (c), wound healing assay, (e), CCK-8 assay (f), or the supernatant were harvested for tube formation assay (d). Data presents as mean ± SEM with three replicates. Ns, not significant; ***, P < 0.001.

**
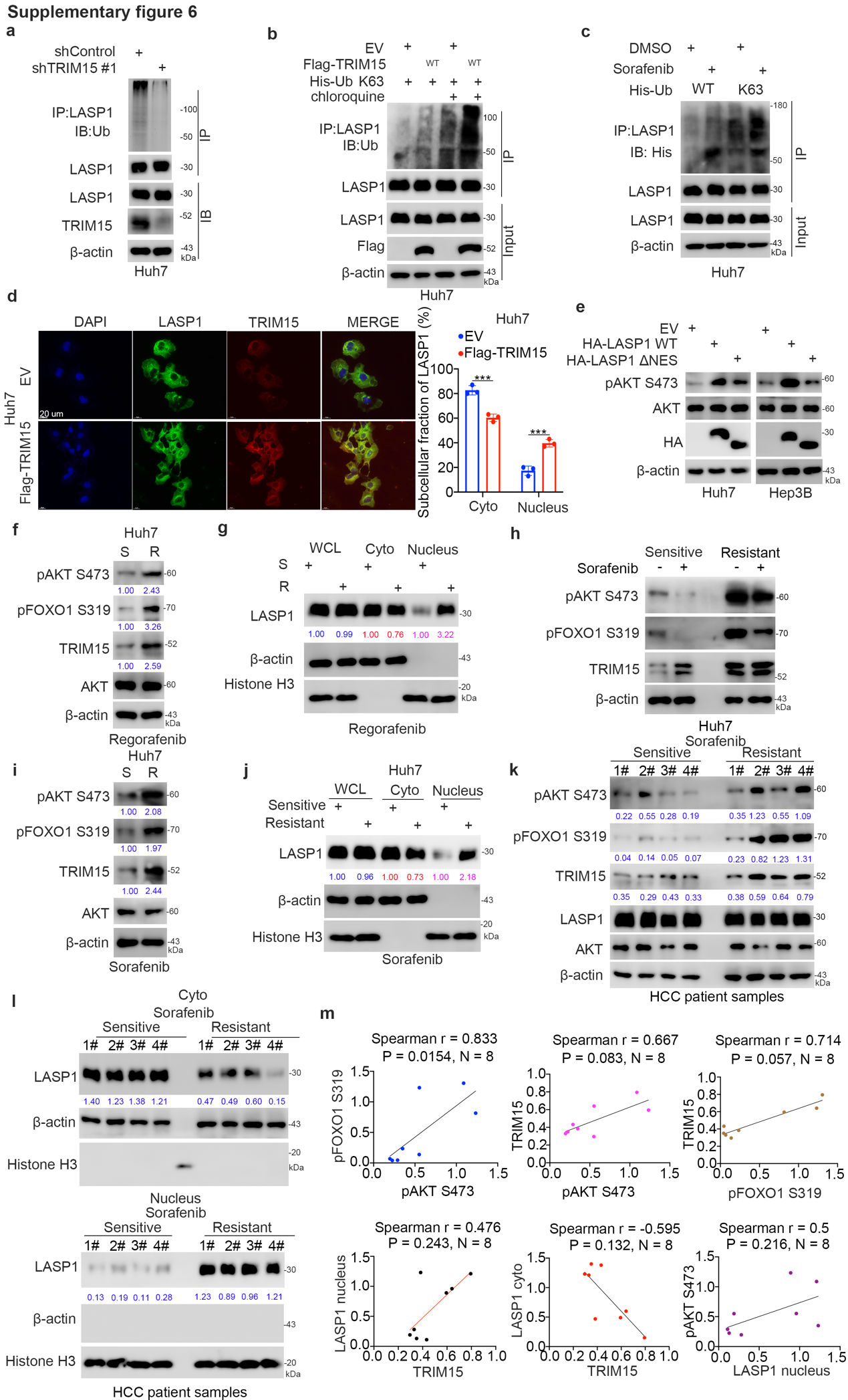
**

**Supplementary figure 6. a,** Huh7 cells were infected with indicated shRNAs for 72 h. Cells were harvested for IP and western blot analysis. **b**, Huh7 cells were infected with indicated shRNAs for 48 h. Then, cells were transfected with indicated plasmids for 24 h. These cells were harvested for IP and western blot analysis before treated with or without chloroquine (5 μM). **c**, Huh7 cells were transfected with indicated plasmids for 24 h. Meanwhile, these cells were treated with or without sorafenib for 24 h. These cells were harvested for IP and western blot analysis. **d**, Huh7 cells were transfected with indicated plasmids for 24 h. These cells were stained with DAPI, LASP1 antibody or TRIM15 antibody and subjected to immunofluorescence assay. Data presents as mean ± SEM with three replicates. ***, P < 0.001. **e**, Huh7 and Hep3B cells were transfected with indicated plasmids for 24 h. Cells were harvested for western blot analysis. **f and g**, the regorafenib sensitive or resistant Huh7 cells were harvested for western blot analysis. The protein levels of pAKT S473, pFOXO1 S319, TRIM15, and LASP1 were quantified by using the Image J software. **h**, the sorafenib sensitive or resistant Huh7 cells were treated with sorafenib (4 μM) for 24 h. Cells were harvested for western blot analysis. **i** **and j**, the sorafenib sensitive or resistant Huh7 cells were harvested for western blot analysis. The protein levels of pAKT S473, pFOXO1 S319, TRIM15, and LASP1 were quantified by using the Image J software. **k-m**, the HCC patient samples were harvested for western blot analysis. The protein levels of pAKT S473, pFOXO1 S319, TRIM15, and LASP1 were quantified by using the Image J software (k and l). The correlation among pAKT S473, pFOXO1 S319, TRIM15, and LASP1 were analyzed, and the correlation coefficient and P values were indicated panel m.

**Table S1. The siRNA and shRNA sequences.**

| siFOXO1 #1 | 5'- CGAACTAGCTCAAATGCTAGT-3' |
| --- | --- |
| siFOXO1 #2 | 5'- GGAGGTATGAGTCAGTATAAC-3' |
| siFOXO1 #3 | 5'- AGTTCATTCGTGTGCAGAATG-3' |
| shTRIM15 #1 | 5'- CACCGCTTCTACAAGATGTCAGACTCGAGTCTGACATCTTGTAGAAGC-3' |
| shTRIM15 #2 | 5'- CACCGGATGTAAAGTGTCAAGAACTCGAGTTCTTGACACTTTACATCC-3' |
| shLASP1 #1 | 5′- CACCGGACCAGATCAGTAACATACTCGAGTATGTTACTGATCTGGTCC-3′ |
| shLASP1 #2 | 5′- CACCGCTACGAGAAGAAGCCCTACTCGAGTAGGGCTTCTTCTCGTAGC-3′ |

**Table S2. The primer sequences for RT-qPCR.**

| Gene（Human） | Forward primer (5′ - 3′) | Reverse primer (5′ - 3′) |
| --- | --- | --- |
| actin beta | ACAGAGCCTCGCCTTTGCC | TGGCCATCTCTTGCTCGAAG |
| TRIM15 | ATGCGGTGACCATTCCCT | TTTCCAGATGATGCGCCAAG |
| LASP1 | TGCAACGCGTGCGCTA | TCAGATGGCCTCCACGTAGT |
| PGF | GCTCAGTCCCTGAAACCCAG | GACACAGGATGGGCTGAACA |

**Table S3. The primer for ChIP-qPCR**

| Gene（Human） | Forward primer (5′ - 3′) | Reverse primer (5′ - 3′) |
| --- | --- | --- |
| TRIM15（FOXO1） | CCTTCTCCCCGCTACAGTAA | GTCCCCTCTTCTGCTCTCCT |


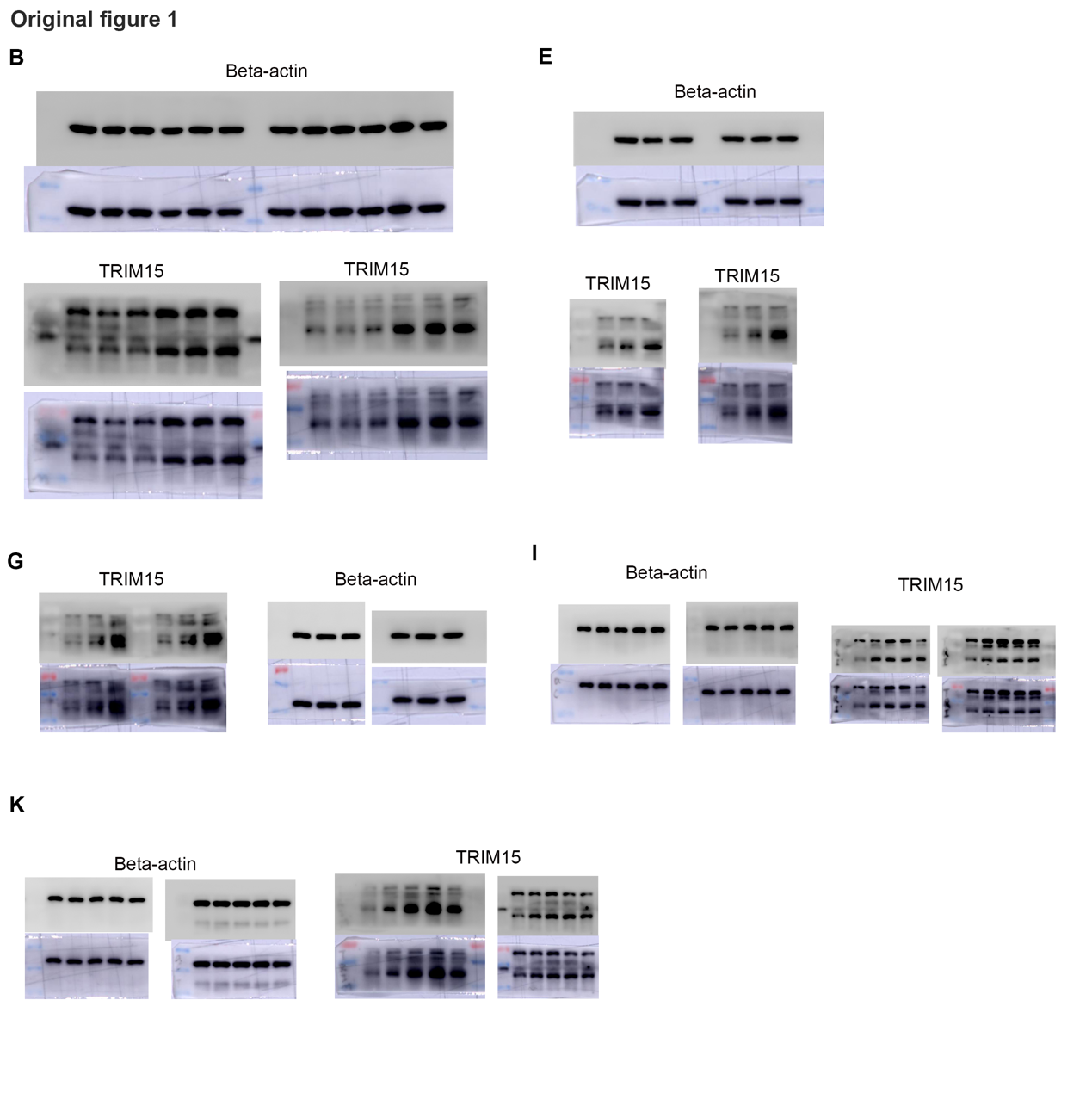

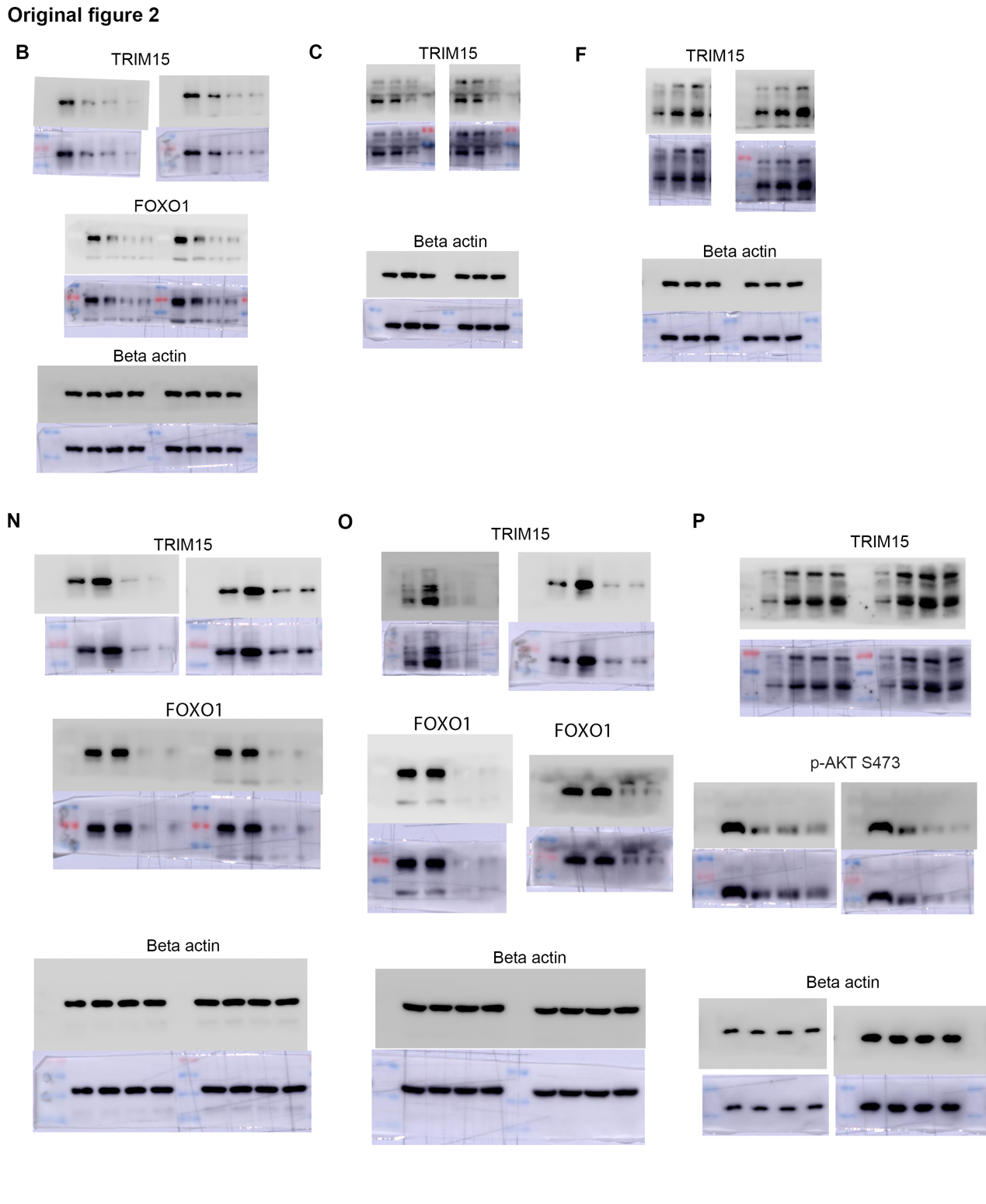

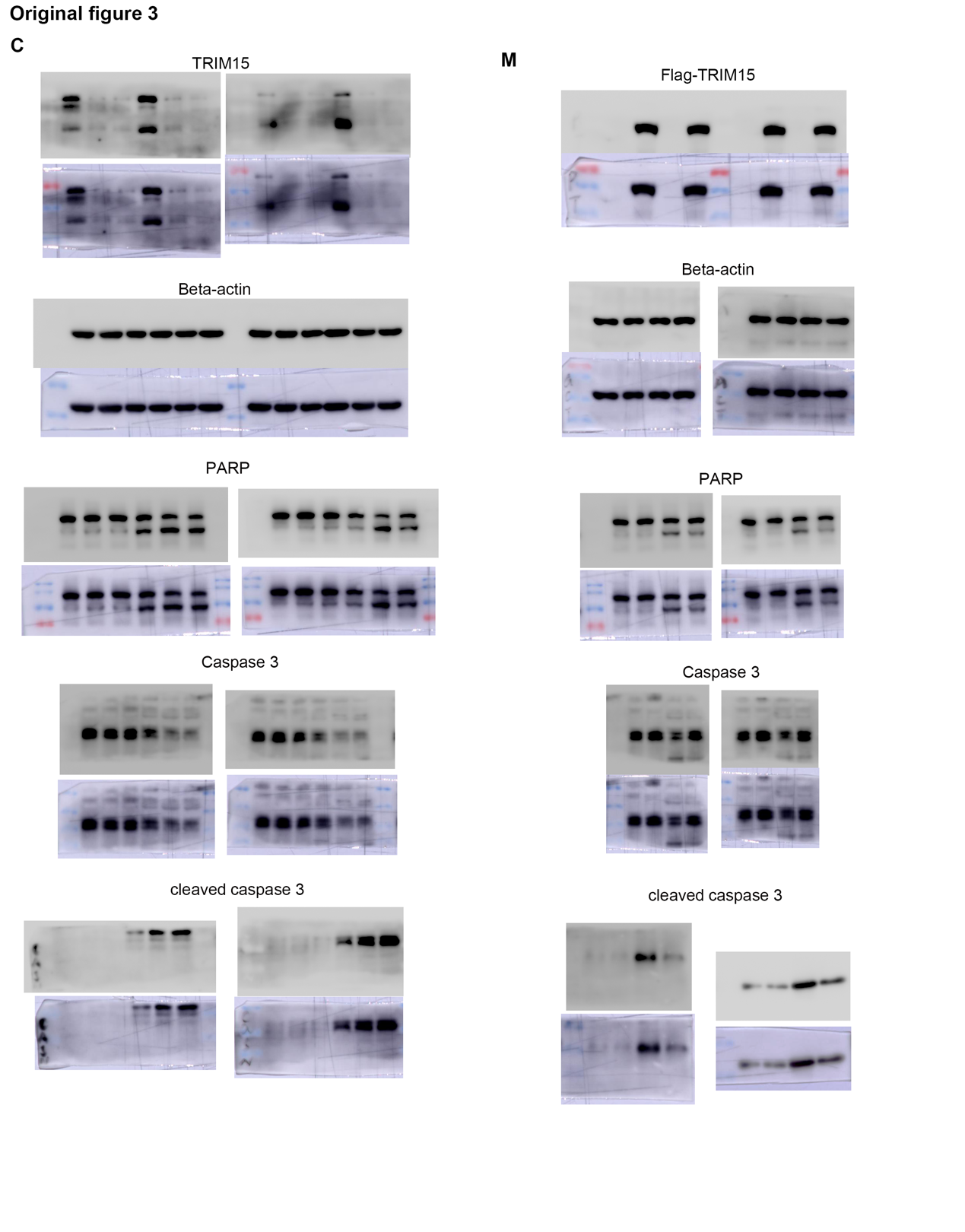

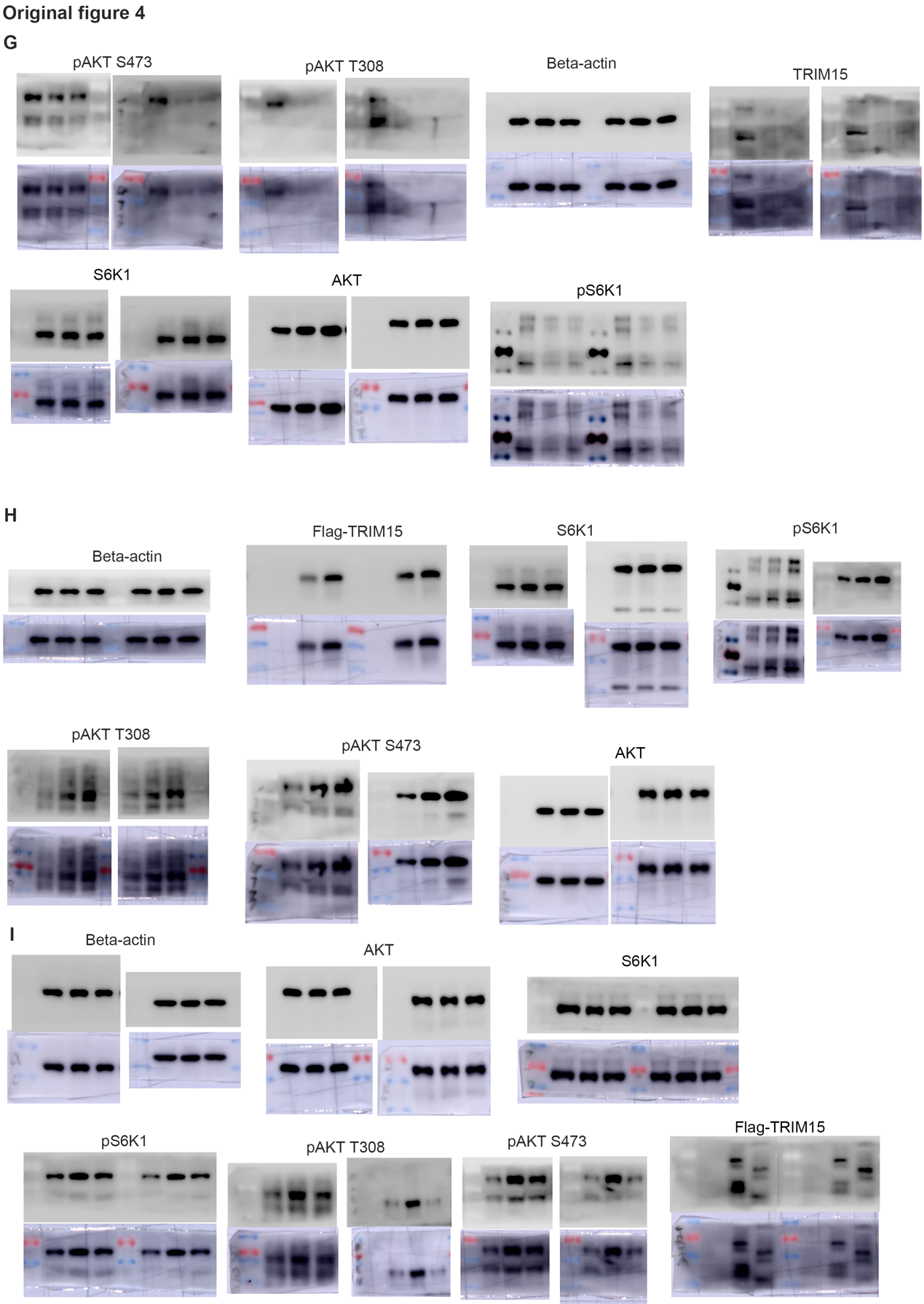

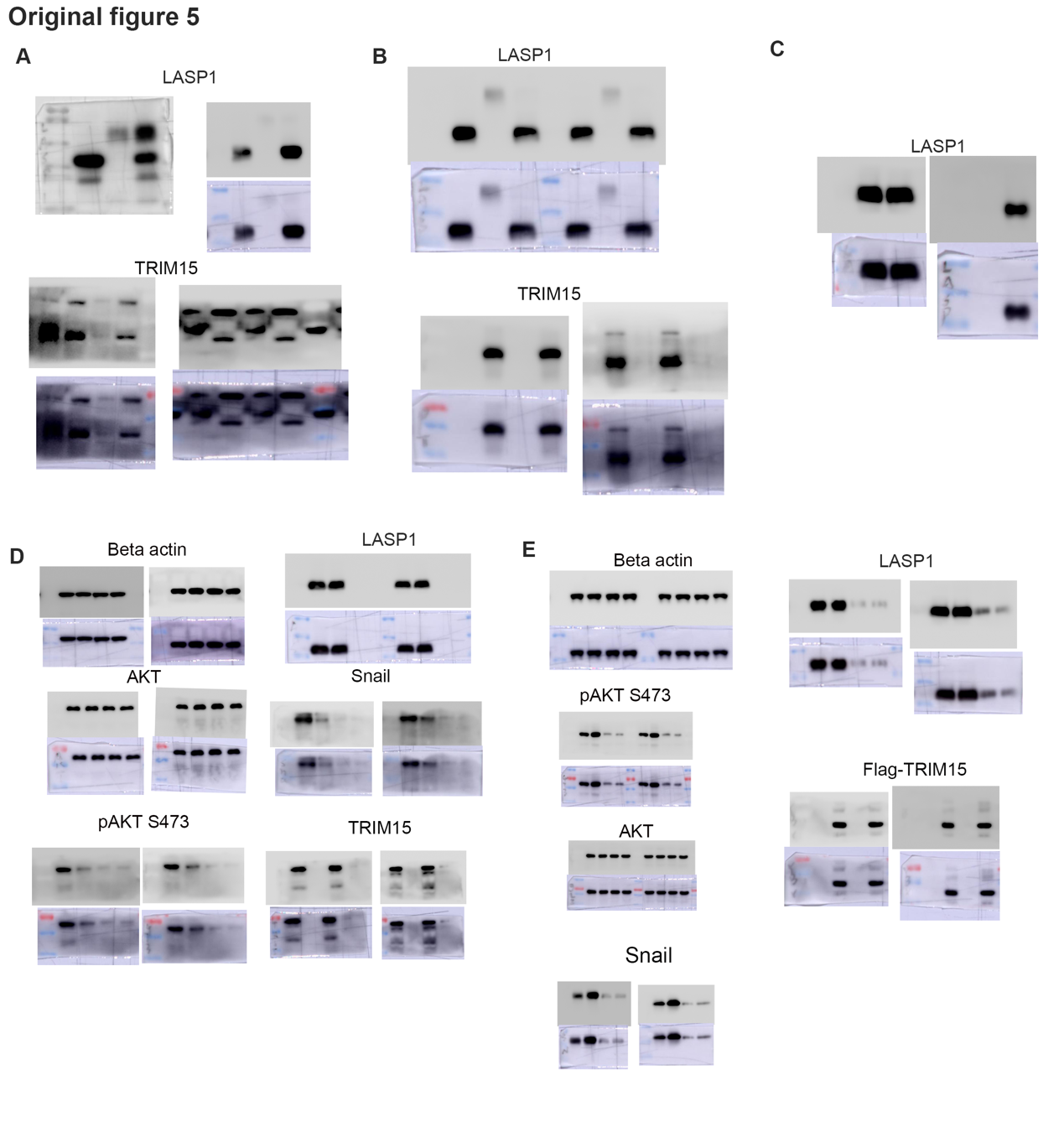

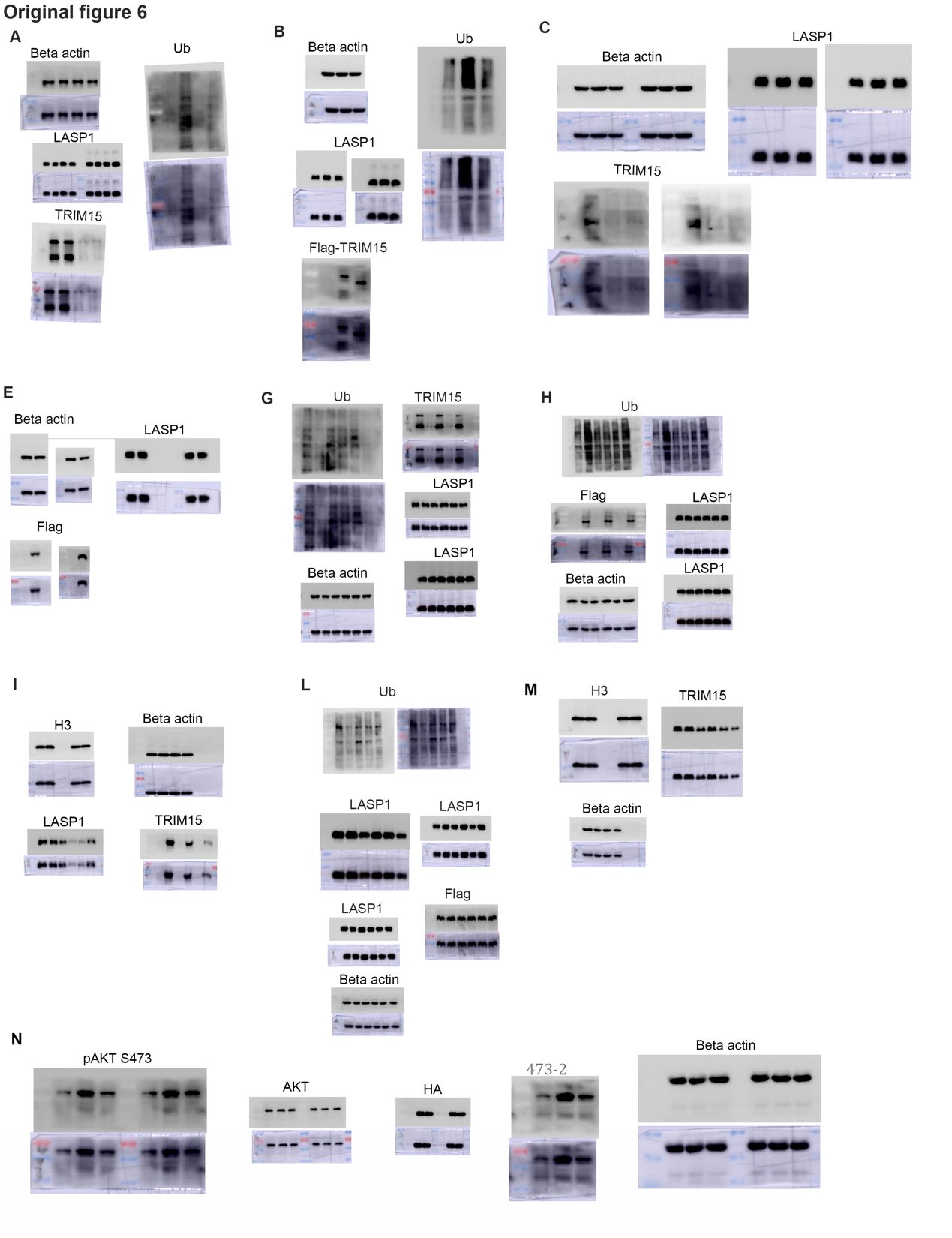


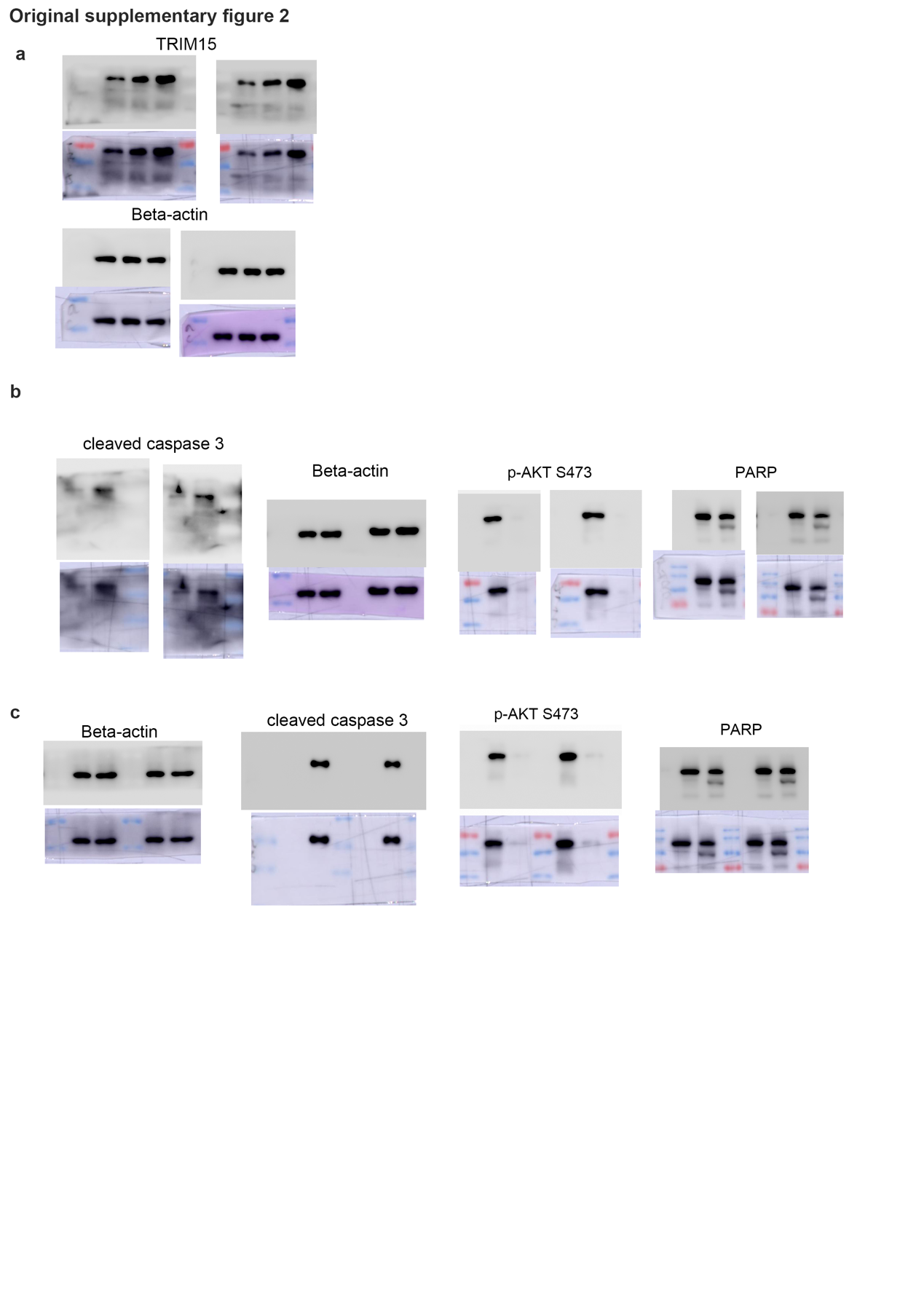


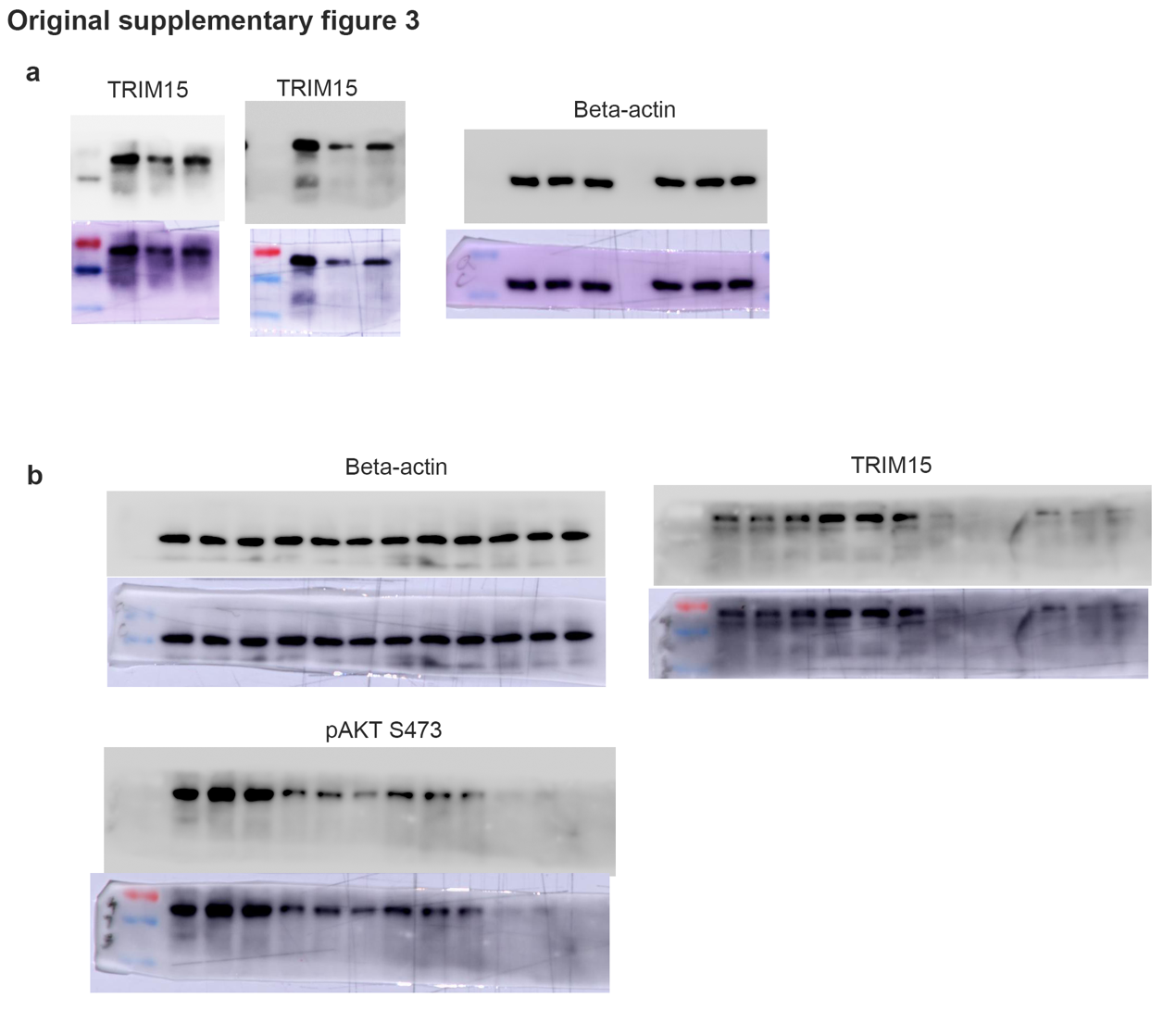


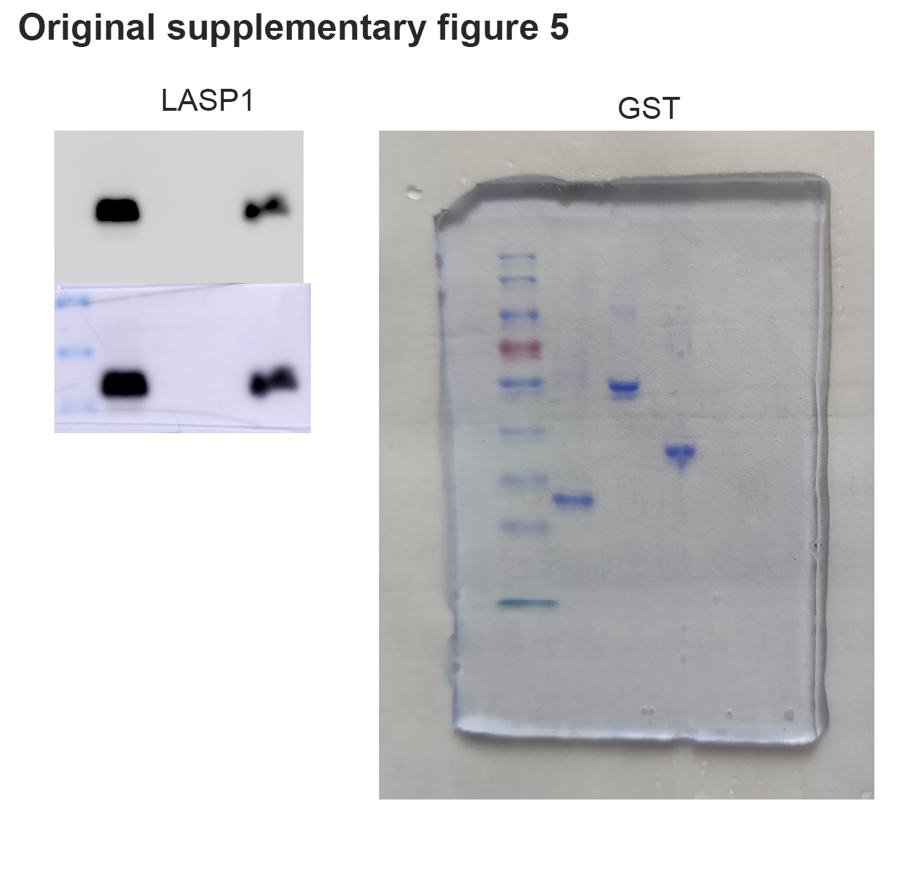

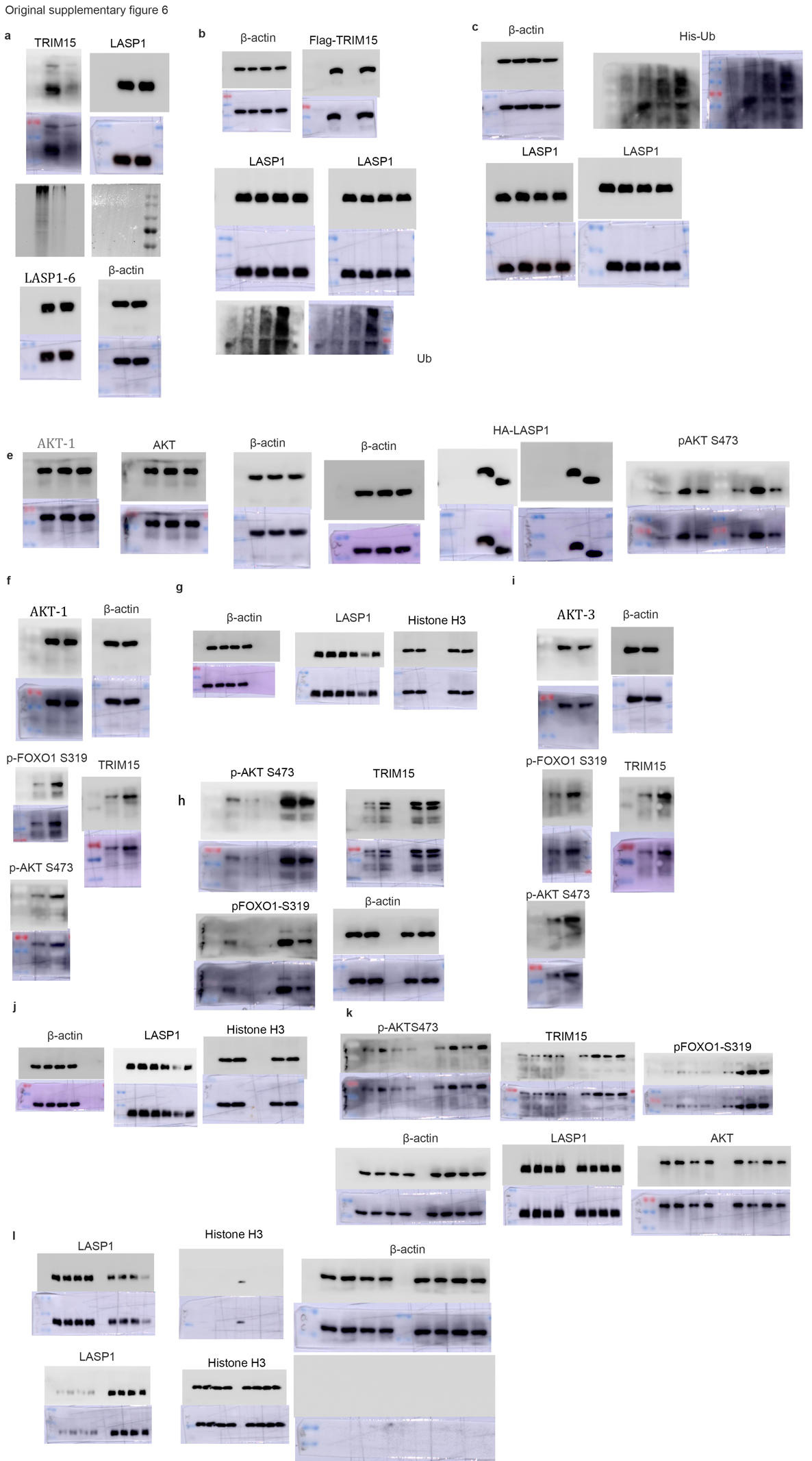

Supplement: Supplementary file 1 — Supplementary information [file 41419_2023_5577_MOESM1_ESM.docx]
