# Supplementary material for: TRIM15 forms a regulatory loop with the AKT/FOXO1 axis and LASP1 to modulate the sensitivity of HCC cells to TKIs
Source: Cell Death Dis. 2023 Jan 20;14(1):47. doi: 10.1038/s41419-023-05577-7 (PMC9859813; doi:10.1038/s41419-023-05577-7)

## Original figure 1

B

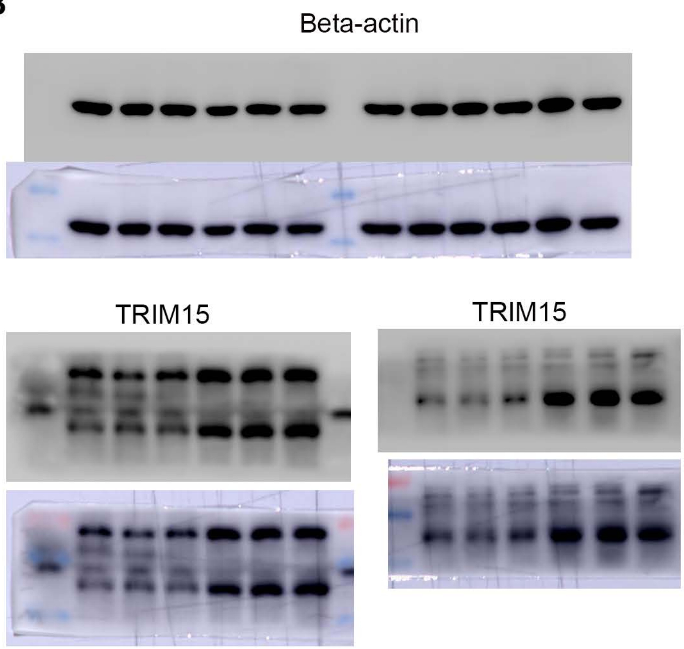


E

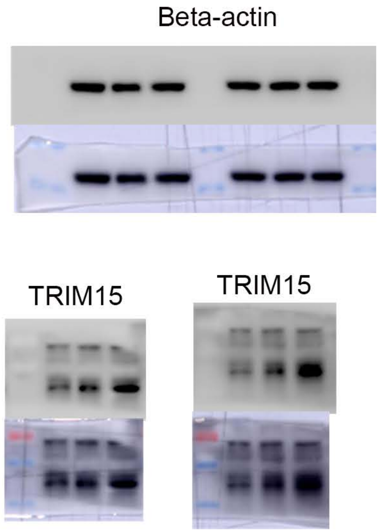


G

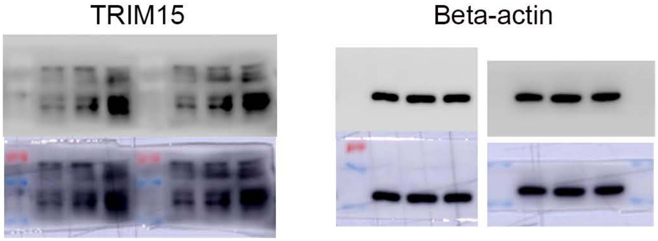


I

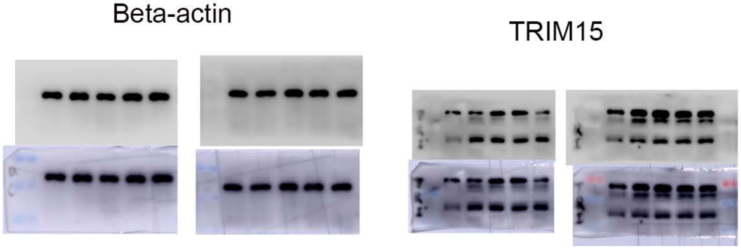


K

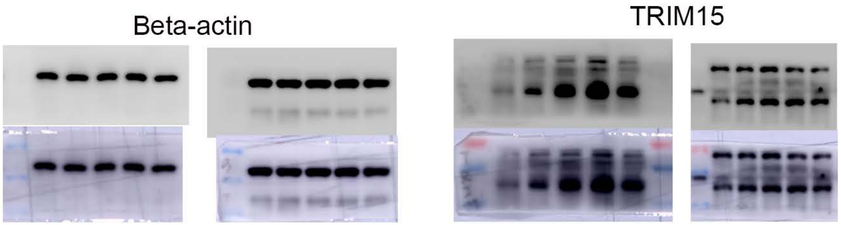

## Original figure 2

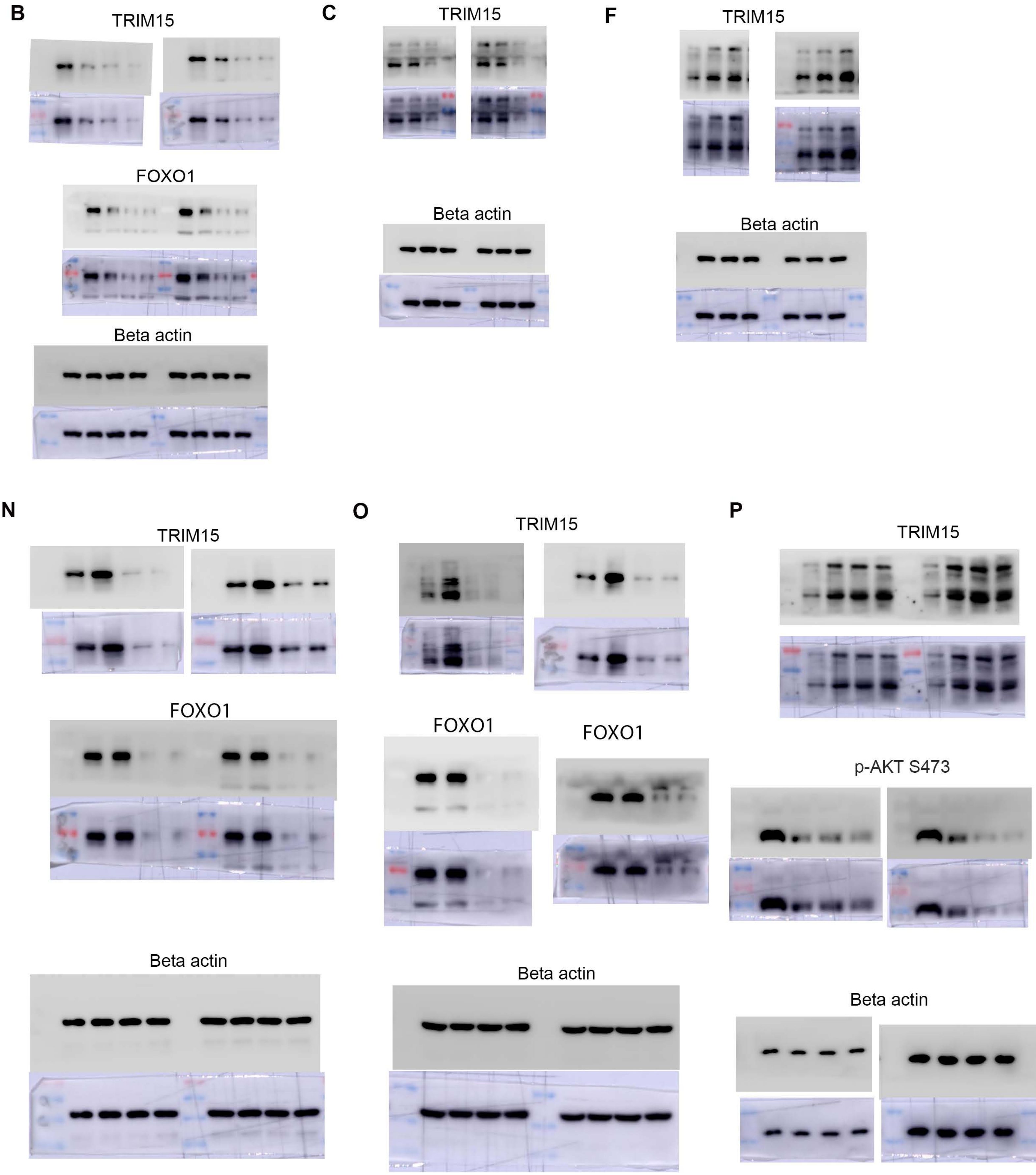

**Original figure 3**

**C**

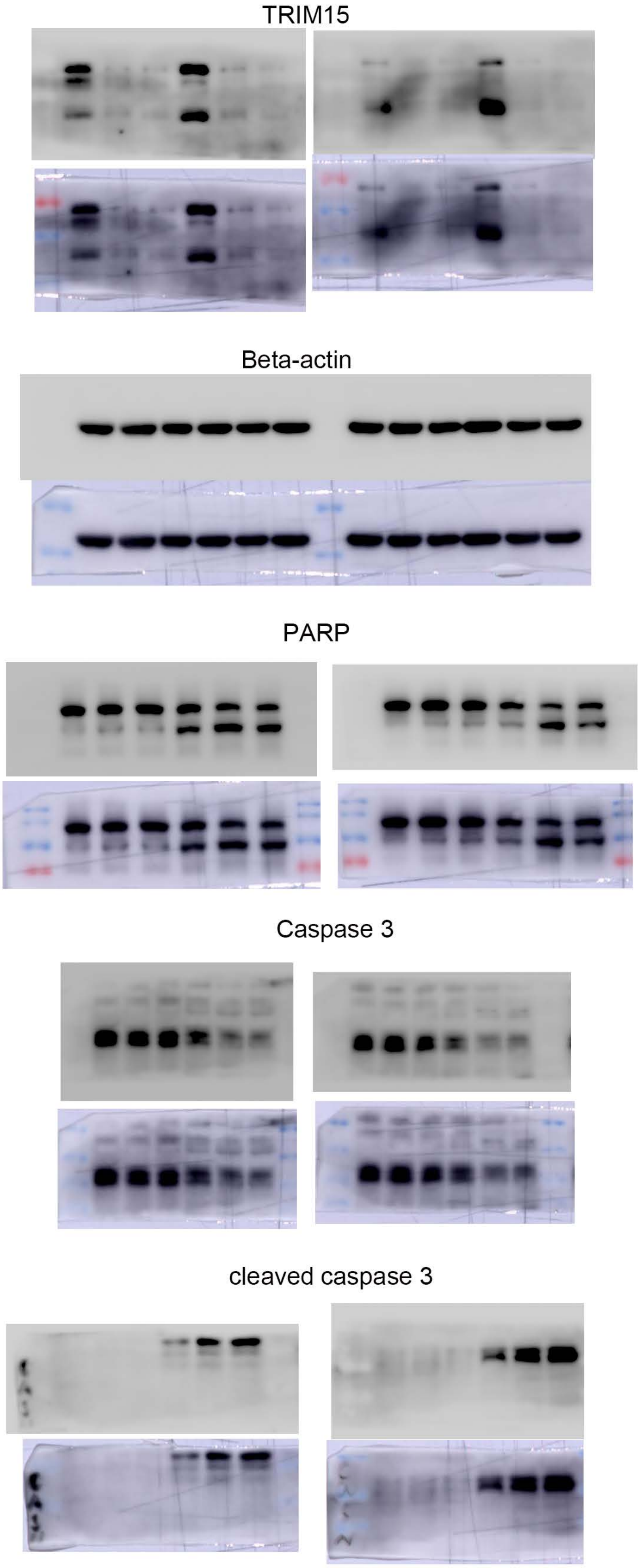


**M**

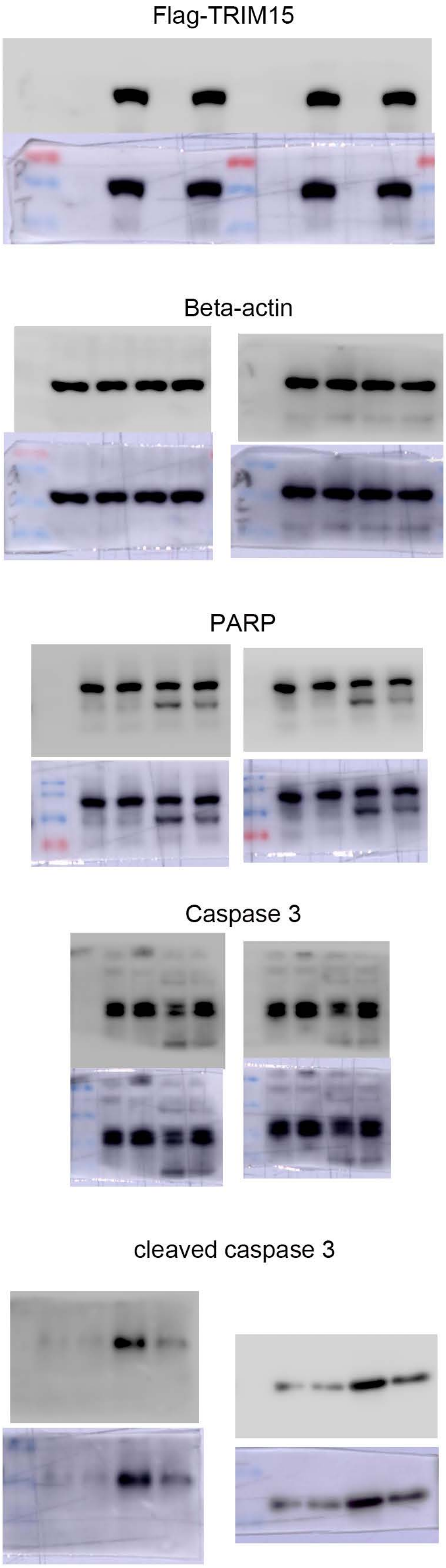

## Original figure 4

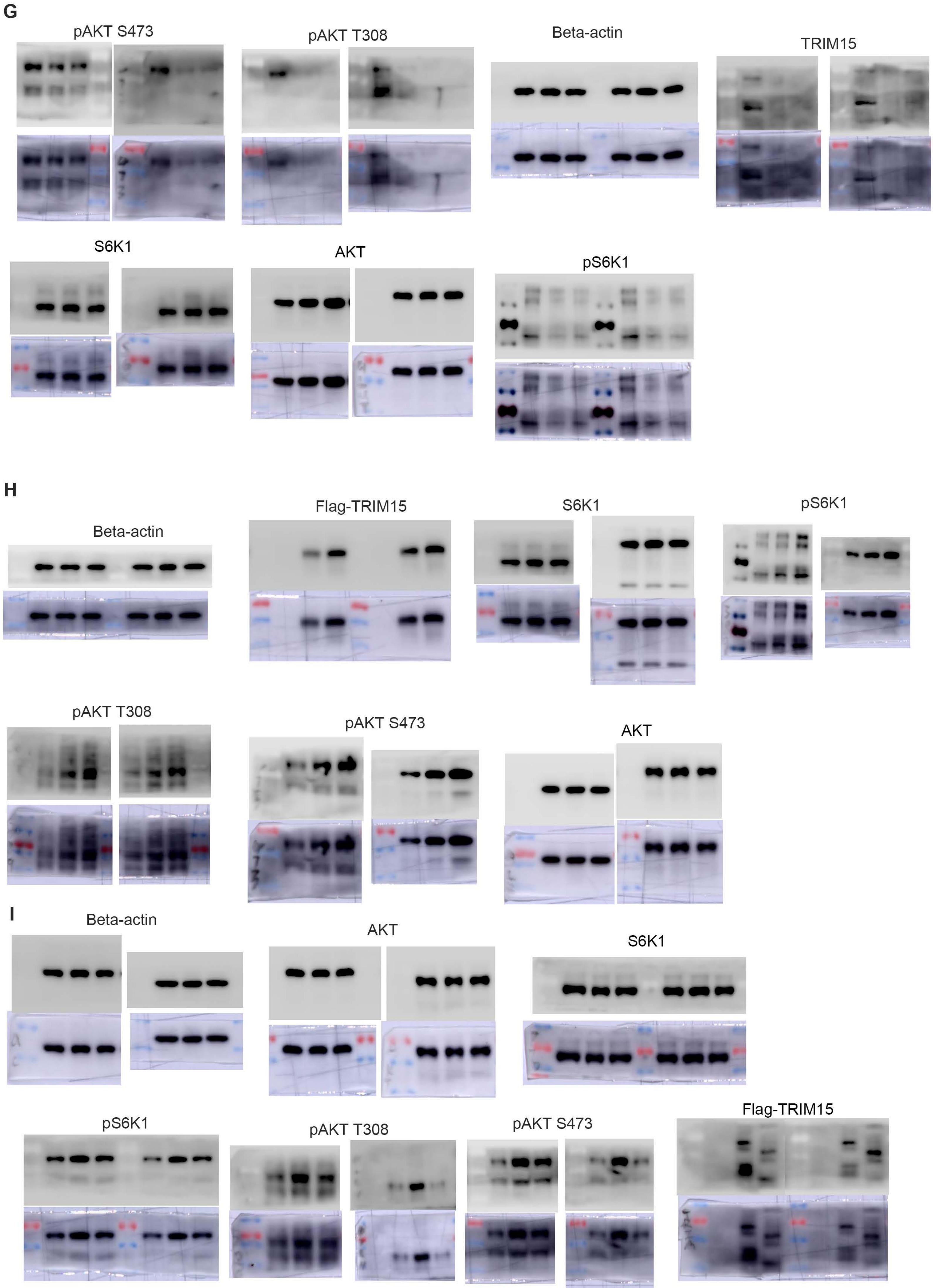

# Original figure 5

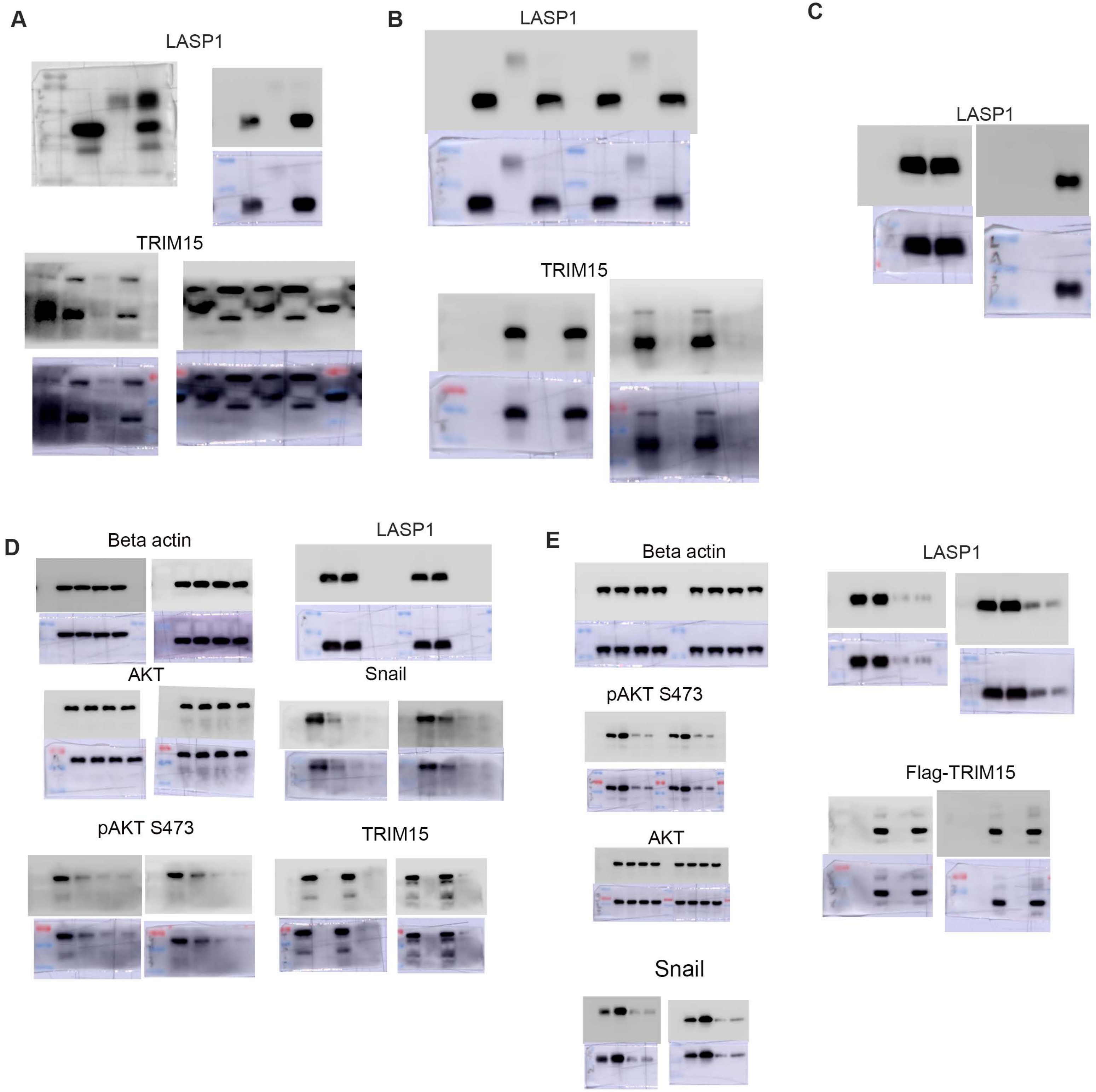

# Original figure 6

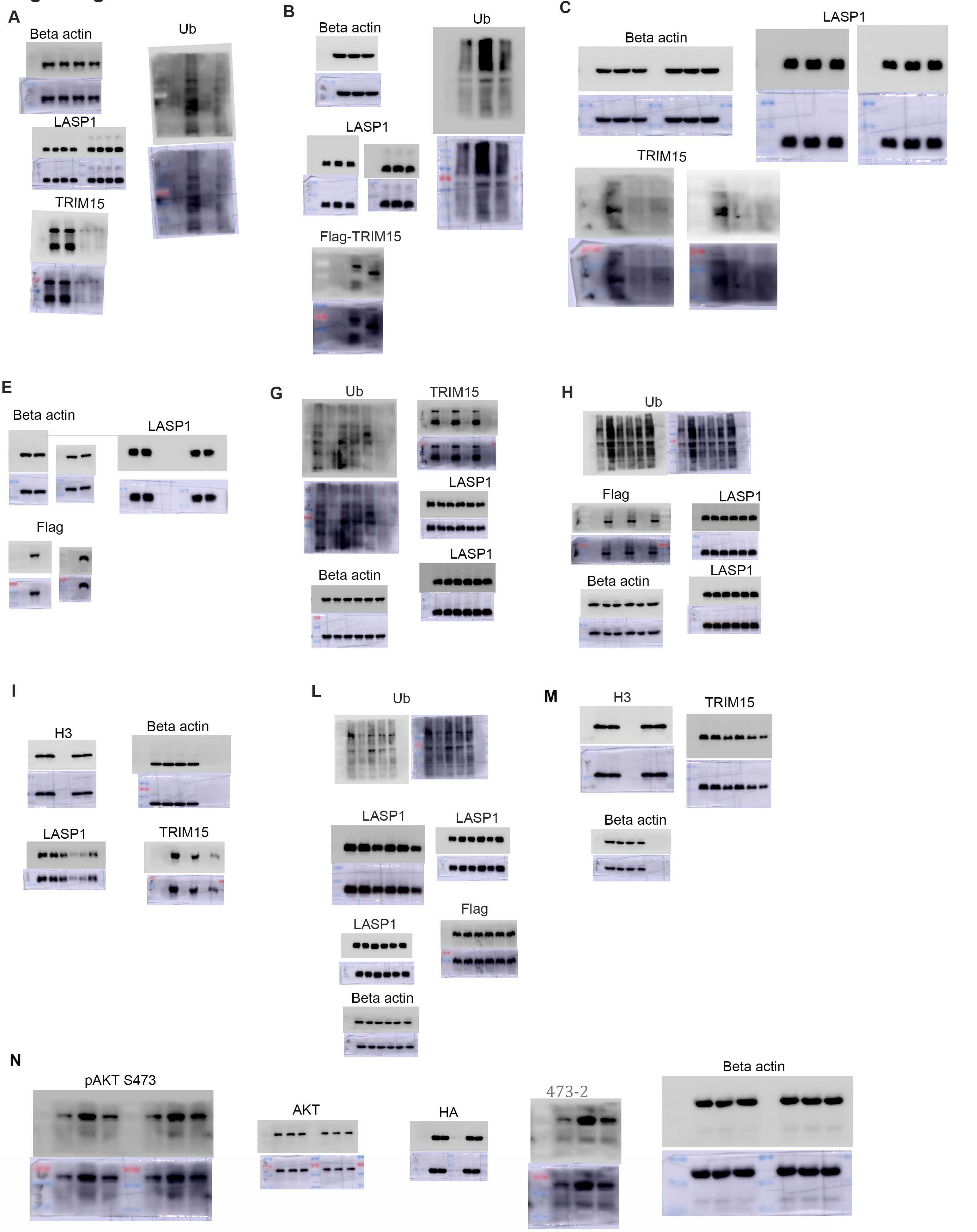

# Original supplementary figure 2

a

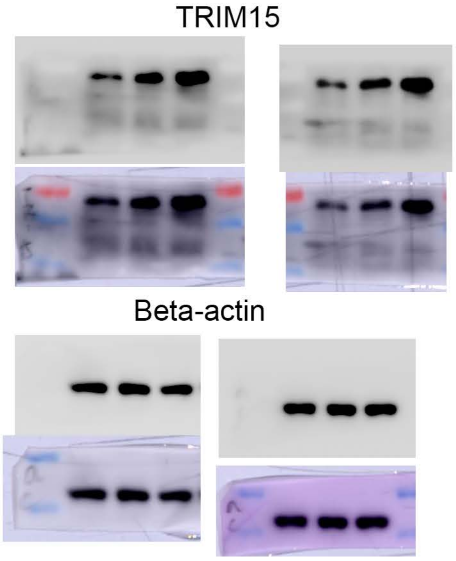


b

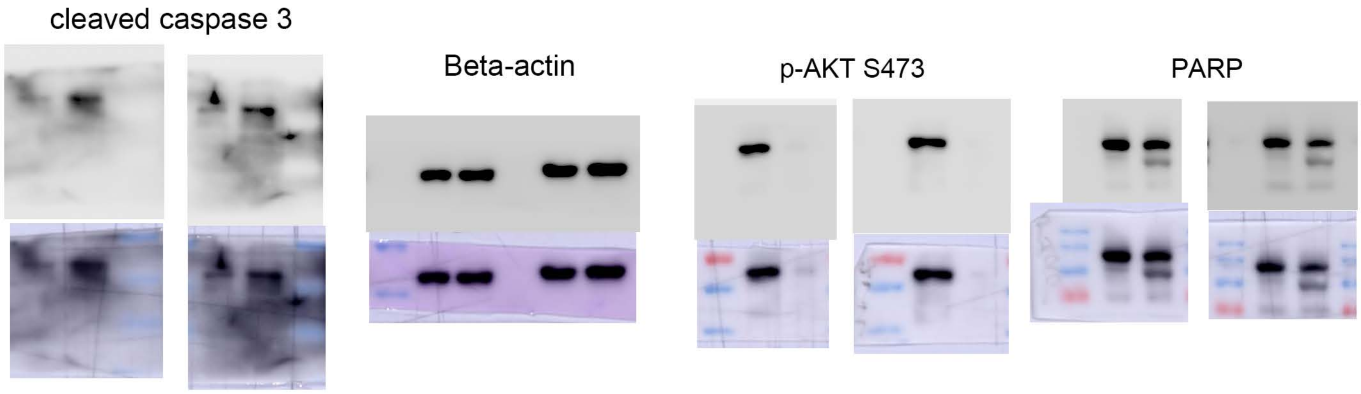


c

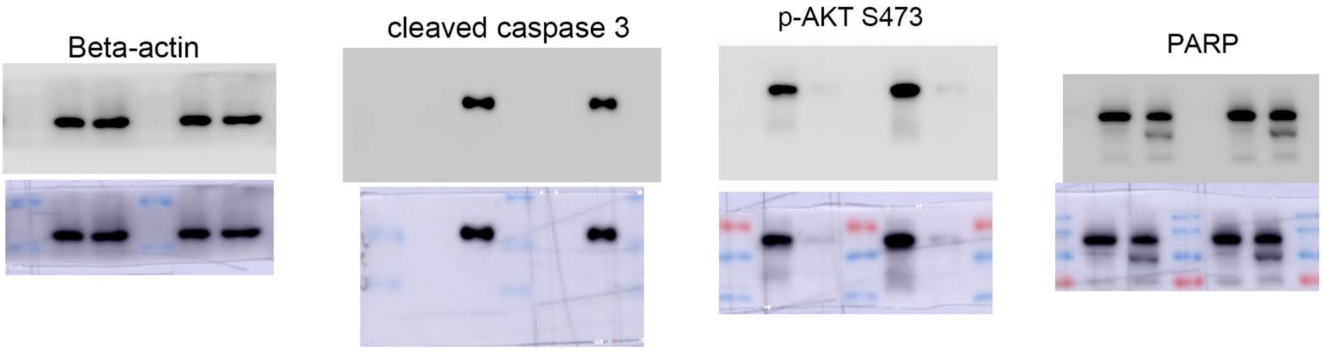

## Original supplementary figure 3

**a**

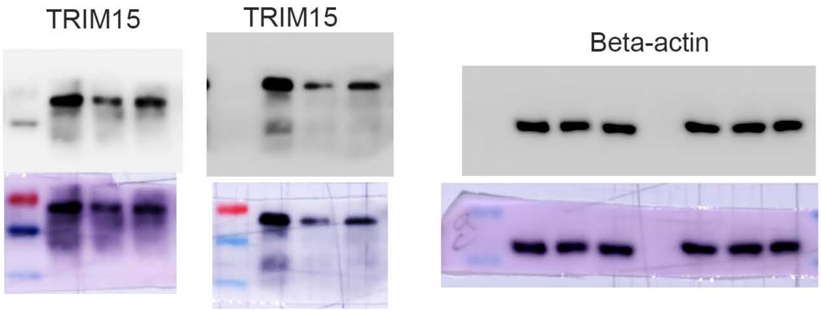


**b**

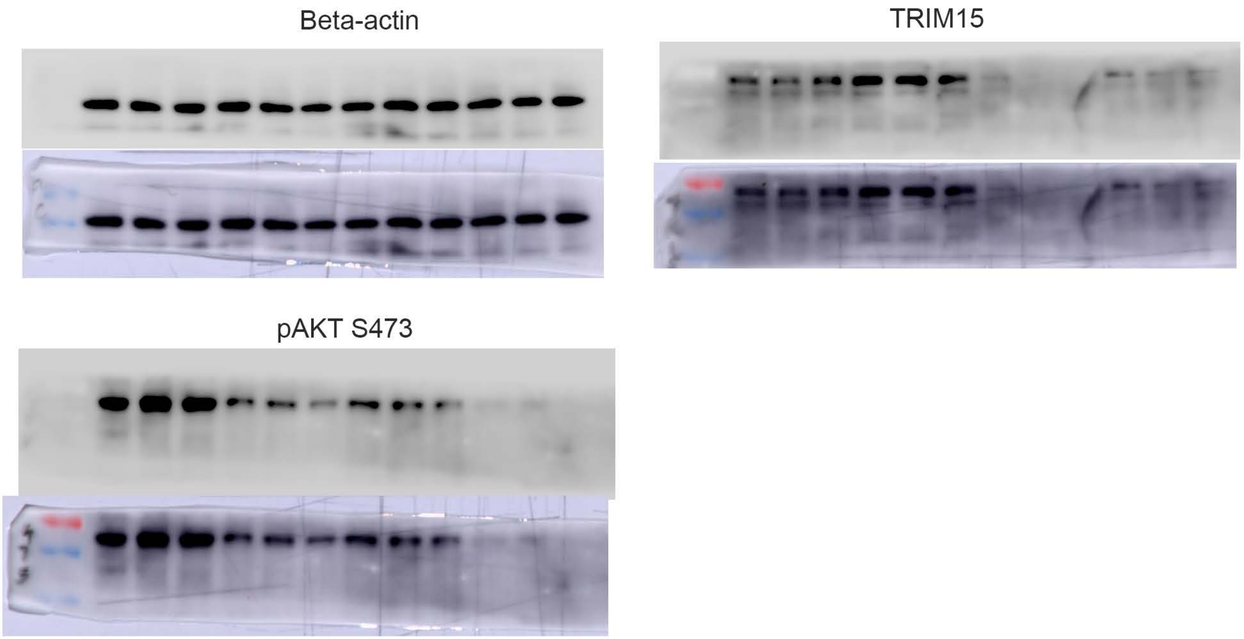

# Original supplementary figure 5

LASP1

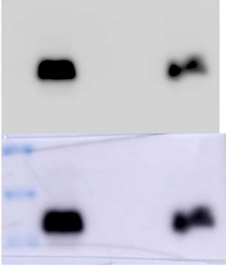

GST

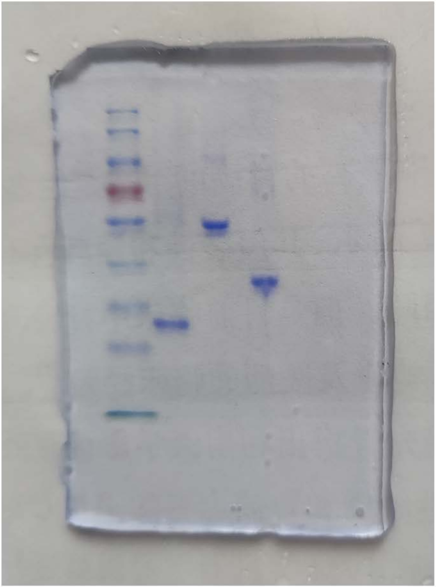

Original supplementary figure 6

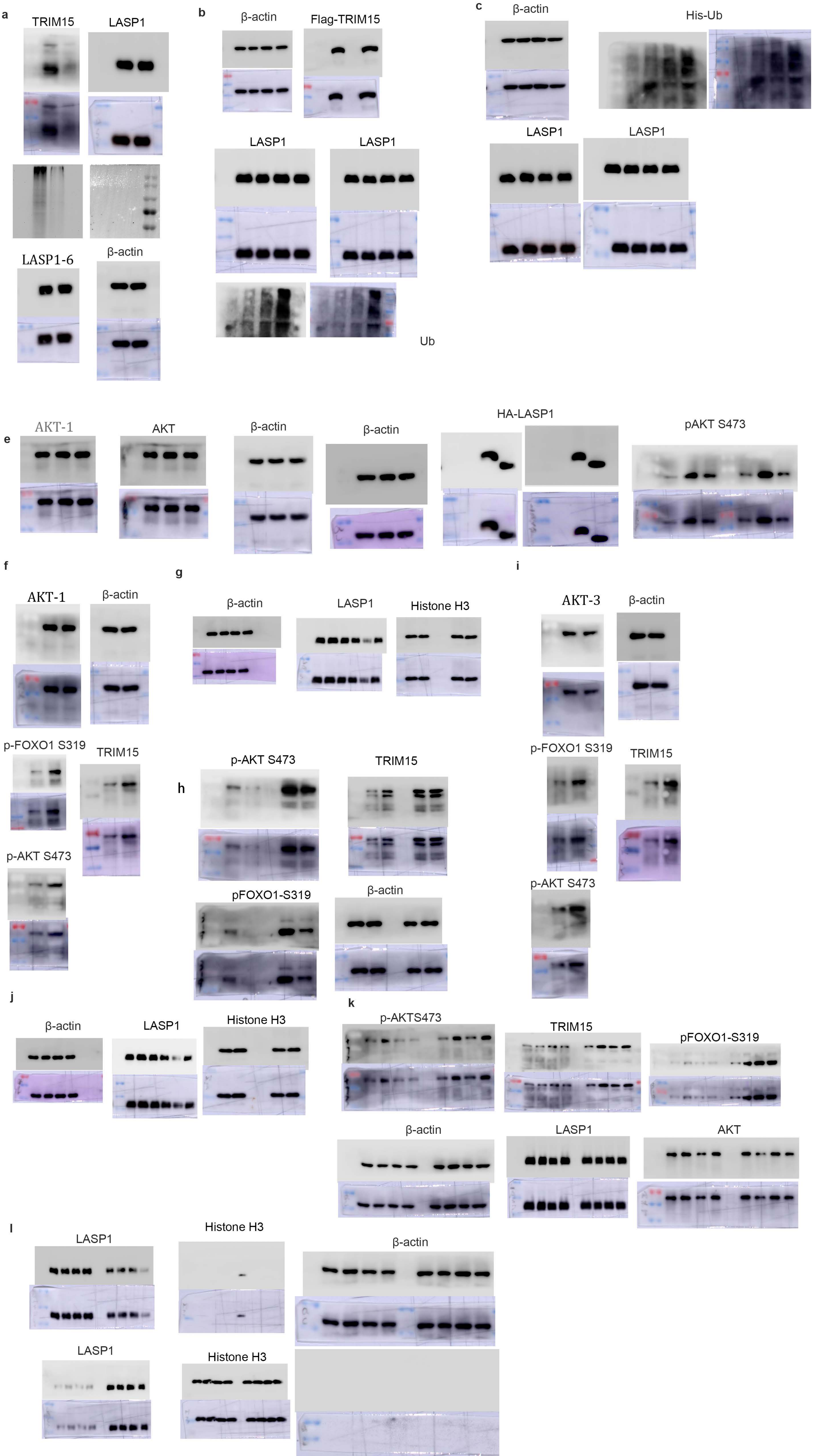

Supplement: Supplementary file 2 — Original Data File [file 41419_2023_5577_MOESM2_ESM.pdf]
